# Development of a novel organoid-based two-dimensional system to model the intestinal host–parasite interface in fasciolosis

**DOI:** 10.3389/fcimb.2026.1886545

**Published:** 2026-09-25

**Authors:** Judit Serrat, Mar Siles-Lucas, Toni Aebischer, Christian Klotz, Javier González-Miguel

**Affiliations:** 1 Laboratory of Helminth Parasites of Zoonotic Importance (ATENEA), Institute of Natural Resources and Agrobiology of Salamanca (IRNASA-CSIC), Salamanca, Spain; 2 Department of Infectious Diseases, Unit for Mycotic and Parasitic Agents and Mycobacteria, Robert Koch Institute, Berlin, Germany

**Keywords:** epithelial barrier function, *Fasciola hepatica*, host–parasite interactions, organoid-derived epithelial monolayers, organoids, quantitative proteomics

## Abstract

**Background:**

The earliest interactions between *Fasciola hepatica* newly excysted juveniles (FhNEJ) and the intestinal epithelium remain poorly understood, partly due to the limited availability of physiologically relevant *in vitro* models. Here, we establish a human duodenum organoid-derived epithelial monolayer (ODM) co-culture system to investigate the early host–parasite interface, providing a controlled experimental platform for analysing interactions between FhNEJ and the intestinal epithelium.

**Methods:**

ODMs representing the human duodenum were co-cultured with either live FhNEJ or a tegument-enriched protein fraction derived from the parasites. The ODM co-culture system was subsequently employed to assess FhNEJ viability and parasite–epithelial interactions together with complementary functional and molecular read-outs. These included measurements of trans-epithelial electrical resistance, fluorescein permeability assays, immunofluorescence analysis of intercellular epithelial junctions, and quantitative proteomic profiling.

**Results:**

The ODM–FhNEJ co-culture system supported sustained parasite viability and enabled direct visualisation of intimate parasite–epithelial interactions. Functional and structural analyses revealed epithelial barrier dysfunction upon exposure to FhNEJ, whilst quantitative proteomics generated an exploratory molecular dataset identifying candidate proteins associated with epithelial junction organisation, immune signalling, cellular stress, and mechanotransduction.

**Conclusion:**

Together, these findings establish human intestinal ODMs as a physiologically relevant experimental platform for investigating the earliest stages of *F. hepatica* infection. By integrating functional, imaging-based and quantitative proteomic analyses within a single experimental framework, this model provides a valuable resource for generating and testing hypotheses regarding host–parasite interactions during early-stage fasciolosis.

## 1 Introduction

The helminth *Fasciola hepatica*, also known as the liver fluke, is the primary etiologic agent of fasciolosis, the most widely distributed food-borne trematodiasis worldwide, accounting for millions of infections in both animals and humans (Fitzpatrick, 2013; Nyindo and Lukambagire, 2015; Mas-Coma et al., 2019). Definitive hosts typically become infected through ingestion of aquatic plants or water contaminated with metacercariae, which release newly excysted juveniles (FhNEJ) upon arrival to the duodenum. Around three to 72 hours after metacercariae excystment, FhNEJ cross the intestinal wall and migrate through the peritoneum until the parasites gain access to the liver and subsequently the major hepatic bile ducts, where adult flukes mature and produce eggs that are shed with faeces (Andrews et al., 2022; Sangster et al., 2022).

As a result of its associated pathology, fasciolosis severely affects animal welfare and leads to millions in economic losses, particularly in livestock. This makes *F. hepatica* one of the most critical helminths impacting the global agricultural and husbandry sectors (Fitzpatrick, 2013; Cwiklinski et al., 2016). Additionally, due to its zoonotic nature, approximately 180 million people are at risk of infection, mainly in tropical regions where people often live under substandard socio-economic conditions. For this reason, the World Health Organization classifies human fasciolosis as a neglected tropical disease of increasing public health concern (Nyindo and Lukambagire, 2015; World Health Organization, 2020). Furthermore, over the past recent years, there has been a steady increase in the number of cases of fasciolosis in which the parasite was resistant to the drug of choice, triclabendazole (TCBZ), raising awareness on the need to find alternative treatment and control strategies (Toet et al., 2014; Kelley et al., 2016; Siles-Lucas et al., 2021). Together, the substantial veterinary burden and the zoonotic nature of fasciolosis, as well as the increasing emergence of TCBZ resistant isolates, highlight the importance of a One Health perspective in the development of sustainable disease-control strategies (Webster et al., 2016). In this context, a deeper understanding of the molecular mechanisms governing host–parasite interactions across different host species would facilitate the identification of novel targets for improved therapeutic and preventive control of fasciolosis (González-Miguel et al., 2021).

Given that most tissue damage caused by *F. hepatica* infection results from the post-intestinal migration of juvenile flukes (Sangster et al., 2022), new approaches to mitigate fasciolosis-associated pathology should focus on preventing the migration of FhNEJ through the intestinal wall. However, the molecular mechanisms underlying *F. hepatica* host invasion, particularly those involved in this early migratory stage, remain poorly understood, largely due to the lack of physiologically relevant and experimentally tractable *in vitro* models that accurately recapitulate the *in vivo* environment (Duque-Correa et al., 2020a; Weiß et al., 2022). For example, primary epithelial cells derived from the mouse small intestine have been co-cultured with FhNEJ to replicate the parasite–gut interface, providing important insights into the molecular responses that are triggered in the intestinal epithelium upon infection (Becerro-Recio et al., 2022b). However, standard culture conditions for intestinal primary cells from mice do not fully reproduce the architecture, polarisation, and the multicellular complexity of the tissue, limiting the physiological relevance of the results in the mammalian context (Duque-Correa et al., 2020a). Likewise, *in vivo* and *ex vivo* animal models employing whole-tissue sections are also available to study the initial stages of *F. hepatica* infection (Becerro-Recio et al., 2022a; López-García et al., 2025), but they do not entirely reflect the human situation and do not comply with current European 3R guidelines to reduce the use of animals for research purposes (European Commission, 2010). Furthermore, analysis at the whole-tissue level dilutes the highly localised epithelial responses occurring at sites of direct parasite contact, hindering precise analysis of parasite-induced changes in intestinal barrier integrity and epithelial function.

Three-dimensional (3D) organoids and related *in vitro* systems have emerged as promising alternatives for the study of host–parasite interactions during infection with clinically-relevant helminths (Duque-Correa et al., 2020b; Smith et al., 2021; Faber et al., 2022; White et al., 2022; Perez et al., 2025; Smith et al., 2026). For instance, co-culture of FhNEJ with spheroids derived from a hepatocyte cell line that recreate the microenvironment of the liver parenchyma has been shown to support parasite survival and growth (Vitkauskaite et al., 2025), offering the opportunity to interrogate the molecular pathways that drive parasite development within the mammalian host. In the context of the intestine, organoids have also been developed from gastrointestinal tissues of different species to investigate host–helminth interactions, providing physiologically relevant models that reproduce the cellular composition of the epithelium and enabling experimental infection with parasitic nematodes of clinical importance (Duque-Correa et al., 2020b; Smith et al., 2021; Faber et al., 2022; White et al., 2022; Perez et al., 2025; Smith et al., 2026). These systems are based on the isolation of intestinal stem cells (SCs) from samples obtained from healthy donors and their subsequent expansion and differentiation into 3D structures that provide a more physiologically relevant system to model the intestinal epithelium *in vitro*. However, the applicability of 3D organoids for the study of helminth biology is limited by the fact that some helminth parasites are too large for microinjection into the organoid lumen (Duque-Correa et al., 2020a), a challenge that can be circumvented by either inverting organoid polarity (Smith et al., 2021; Chapuis et al., 2025) or by culturing intestinal SCs in a two-dimensional (2D) conformation using transwells, with the apical compartment readily-available for parasite stimulation (Duque-Correa et al., 2022; Holthaus et al., 2022). In fact, 2D organoid-derived epithelial monolayers (ODMs) obtained from adult SCs of healthy duodenum faithfully replicate the cellular composition of the tissue of origin as well as its electrophysiological and permeability properties (Weiß et al., 2022), providing a physiologically relevant and experimentally tractable platform to study host–parasite interactions at the intestinal epithelium (Holthaus et al., 2022). However, the suitability of human duodenum ODMs for co-culture with FhNEJ and the study of early host–parasite interactions has not yet been explored.

In this study, we establish human duodenum ODMs as a novel experimental system for investigating the interactions between FhNEJ and the intestinal epithelium. We assessed the ability of the system to support parasite viability, visualised intimate parasite–epithelial interactions, and evaluated whether the system could support functional, structural, and molecular readouts. These included measurements of electrophysiological and permeability properties of the epithelium, analysis of intercellular junction integrity, and quantitative proteomic profiling, which can be employed as an exploratory tool to identify candidate host responses and generate hypotheses for future mechanistic investigations.

## 2 Results

### 2.1 Establishment of a human duodenum ODM–FhNEJ co-culture model

First, ODMs were produced from 3D SC-enriched organoids derived from the human duodenum using standard procedures to replicate the natural niche of FhNEJ (**Supplementary Figure 1**). After ODM differentiation following established protocols (Holthaus et al., 2022; Warschkau et al., 2022), the resulting monolayers have previously been shown to comprise a polarised intestinal epithelium enriched in absorptive enterocyte-like cells together with Paneth cells and proliferative cells, consistent with the retention of a stem/progenitor cell compartment. Mucus-producing goblet cells and enteroendocrine cells have not been detected in this system (Holthaus et al., 2022; Warschkau et al., 2022). Freshly excysted FhNEJ were obtained *in vitro* from commercially available metacercariae and added to the ODMs at days 11 or 12 after seeding. Live time-lapse imaging and visual inspection revealed that FhNEJ were alive and highly motile for at least 48 h after addition to the ODMs (**Supplementary Video 1**), indicating the compatibility of the ODM system with FhNEJ viability.

Immunofluorescence analysis (IFA) combined with confocal microscopy enabled direct visualisation of the intimate interaction between FhNEJ and the intestinal epithelium within the ODM co-culture system. This analysis showed that FhNEJ established direct contact with the epithelial surface (**Figure 1A**) and employed their ventral sucker to stably attach to it (**Figure 1B**). The strength of this attachment was evidenced by the retention of the parasites throughout the multiple washing steps required for immunostaining. Interestingly, a small fraction of FhNEJ (1–3 per transwell out of 50 added) were located beneath the epithelial layer, with their ventral sucker remaining in direct contact with the intestinal epithelial cells (**Figure 1C**; **Supplementary Video 2**). This observation illustrates the ability of the model to capture parasite localisation relative to the epithelial monolayer, thereby enabling future mechanistic investigations of parasite migration through the intestine during early stage infection.

**Figure 1:**
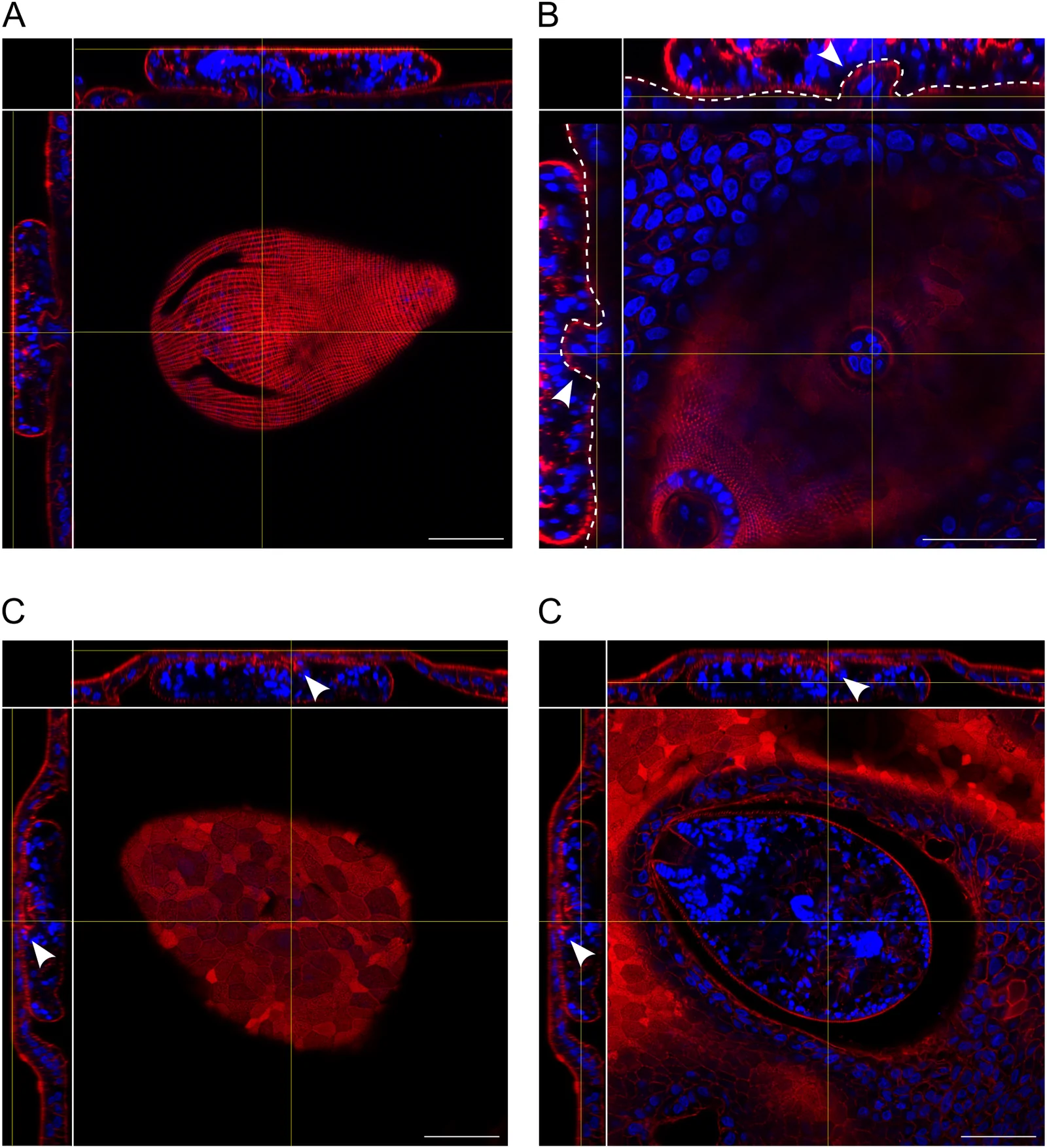
Incubation of ODMs with live FhNEJ. Forty-eight hours after addition of FhNEJ, ODMs were fixed, stained for IFA and visualised by confocal microscopy. Phalloidin (red) was used to highlight actin filaments present in the apical border of epithelial cells and the surface and internal muscle fibres of FhNEJ. DAPI (blue) was used to visualise nuclear DNA. Specimens were visualised in a Leica Stellaris 8 confocal microscope using the 40× objective. **(A, B)** The FhNEJ were mostly found on the apical side of the epithelium, in direct contact with epithelial cells and attached to them via their ventral sucker (arrowheads). In B, the interface between the parasite and the epithelial layer has been underlined with a white dashed line. **(C)** Occasionally, some FhNEJ were recovered under the epithelial layer (left panel, apical plane of the projection; right panel, middle plane of the projection). In panels **(B, C)**, arrowheads indicate the FhNEJ ventral sucker. Scale bars, 50 µm.

### 2.2 Application of the ODM co-culture model to study epithelial responses to live FhNEJ

To determine the suitability of the ODM–FhNEJ co-culture system for studying epithelial barrier function during parasite engagement, epithelial integrity was evaluated using complementary functional and structural readouts. The formation of a functional epithelial barrier in ODMs was shown by a constant increase in trans-epithelial electrical resistance (TEER) overtime that stabilised 11–12 days after establishment of the monolayers (**Figure 2A**), consistent with previous reports (Holthaus et al., 2022). At this time, ODMs were co-cultured with either 5 FhNEJ or 50 FhNEJ for 48 h and epithelial barrier function and permeability were assessed by TEER measurements and fluorescein flux assays. Untreated ODMs served as negative controls, and ODMs treated with the apoptosis-inducer staurosporin served as positive controls for epithelial barrier breakdown (Holthaus et al., 2022).

**Figure 2:**
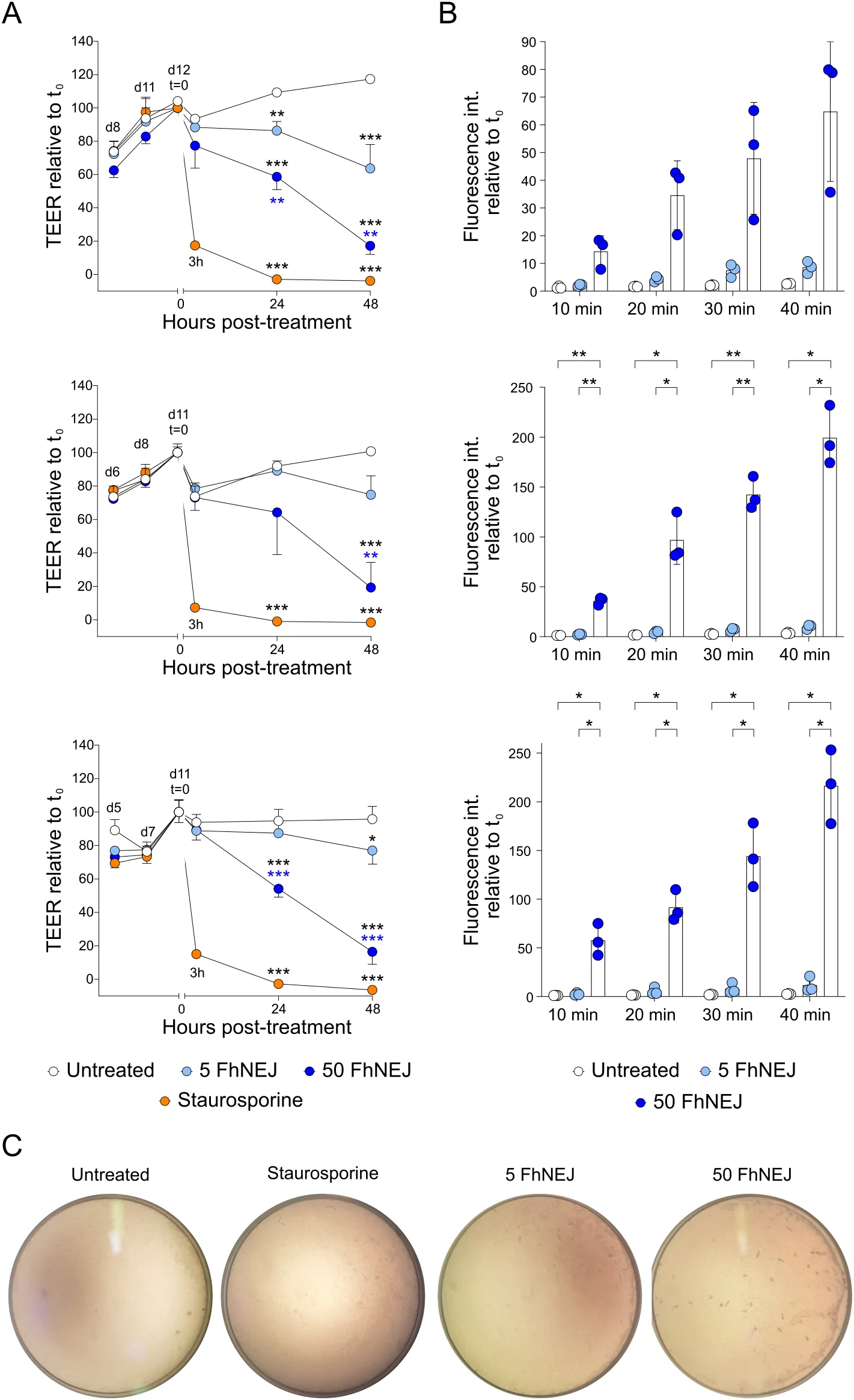
Incubation of ODMs with FhNEJ results in a dose- and time-dependent decrease in epithelial barrier function. **(A)** Dose- and time-dependent decrease in TEER after stimulation with FhNEJ. Treatment of ODMs with the apoptosis-inducer staurosporine (2 µM) served as a positive control for epithelial barrier breakdown. Data points indicate the mean of three technical replicates ± SD. Black asterisks indicate significant differences between the indicated groups and the untreated condition, and blue asterisks indicate significant differences between ODMs treated with 5 FhNEJ and 50 FhNEJ (**p<0.01; ***p<0.001; one-way ANOVA). **(B)** Fluorescein flux into the basal compartment of ODMs was measured 48 hours after infection at 10, 20, 30, and 40 minutes following fluorescein addition to the apical compartment. Results for staurosporine-treated samples are not shown as they displayed maximal fluorescence intensity (between 100× and 300× higher than t_0_) at the earliest timepoint. Bars indicate the mean of three technical replicates ± SD, and asterisks indicate significant differences between the indicated groups (*p<0.05; **p<0.01; two-way ANOVA). In panels **(A, B)**, results from three independent experiments are shown. Each experiment was performed using ODMs generated from a successive passage of the duodenal 3D organoids and FhNEJ obtained from an independent sub-batch of freshly excysted *F. hepatica* metacercariae. **(C)** Representative stereoscope images of ODMs incubated with 5 FhNEJ, 50 FhNEJ, or left untreated 48 hours after addition of the parasites. Images from two additional independent experiments are shown in **Supplementary Figure 2**.

The co-culture model enabled the detection of dose-dependent alterations in epithelial barrier function following exposure to live FhNEJ. TEER values progressively decreased after parasite addition, with measurable changes detected after 24 h and becoming more pronounced at the higher parasite dose (**Figure 2A**). Accordingly, epithelial permeability assessed by fluorescein flux to the basolateral compartment at the end of the infection time course (48 h after FhNEJ addition) was proportional to the number of parasites added to the ODMs (**Figure 2B**). In addition, microscopic examination revealed partial detachment of the epithelial monolayer from the edges of the transwell membrane in cultures incubated with 50 FhNEJ (**Figure 2C**; **Supplementary Figure 2**).

To further characterise the structural integrity of the epithelial barrier within the co-culture system, the localisation and abundance of the prototypic tight junction proteins zonula occludens 1 (ZO-1) and claudin-1 (CLDN-1) were examined by IFA and immunoblotting. Results showed that ODMs incubated with 50 FhNEJ displayed a marked reduction in ZO-1 signal at the apical domain of epithelial cells (**Figure 3A**; **Supplementary Figure 3**), which was accompanied by a modest but significant reduction in total ZO-1 abundance as determined by immunoblotting (**Figures 3B, C**). In contrast, although CLDN-1 protein abundance was also slightly reduced (**Figures 3B, C**), its localisation at apical tight junctions remained largely preserved (**Figure 3A**; **Supplementary Figure 3**). Together, these complementary approaches demonstrate that the ODM–FhNEJ co-culture model supports multiple functional readouts for evaluating epithelial barrier integrity during interaction with live FhNEJ.

**Figure 3:**
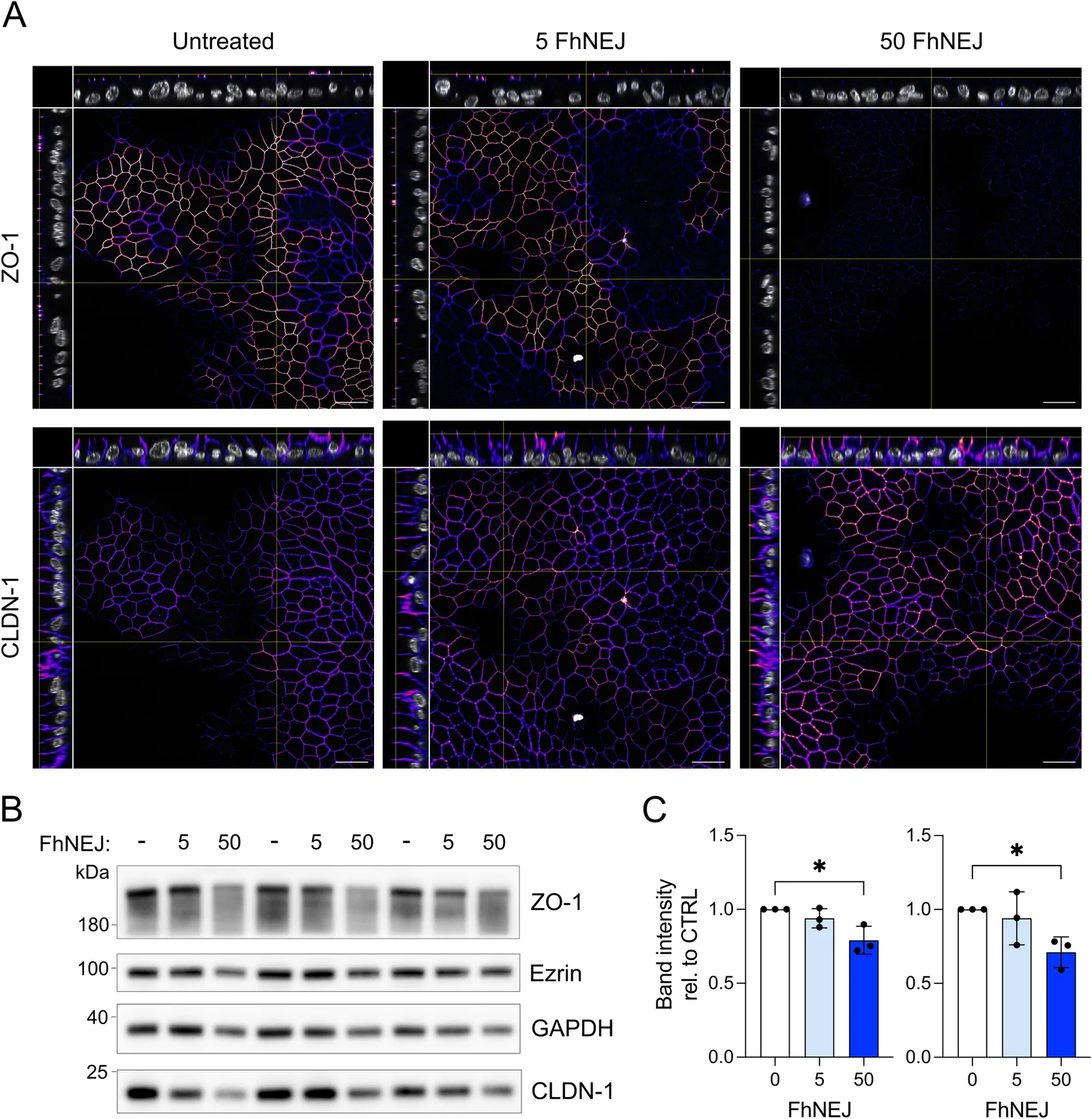
Incubation of ODMs with high doses of FhNEJ affects ZO-1 localisation at the apical domain of intestinal epithelial cells. **(A)** Orthogonal stacks of representative IFA images of tight junction proteins ZO-1 and CLDN-1 48 hours after addition of FhNEJ. Images show a single channel displayed with colour-coded intensities. DAPI is shown in greyscale. Specimens were visualised in a Leica Stellaris 8 confocal microscope using the 63× objective. Scale bars, 20 µm. Orthogonal stacks of IFA images from two additional independent experiments are shown in **Supplementary Figure 3**. **(B)** Representative immunoblot analysis of tight junction proteins ZO-1 and CLDN-1 48 hours after addition of FhNEJ. Results are shown for three independent experiments. Ezrin and glyceraldehyde phosphate dehydrogenase (GAPDH) served as loading controls. The second immunoblot replicate, uncropped immunoblot images, and total protein staining with Ponceau S are shown in **Supplementary Figures 5A–E, G**. **(C)** Quantification of ZO-1 and CLDN-1 band intensities. Individual data points represent three independent experiments. For each immunoblot, target protein abundance was independently normalised to GAPDH and Ezrin, and the two normalised values were averaged. Values obtained from two technical immunoblot replicates were then averaged to generate a single value for each biological replicate. Bars indicate the mean ± SEM of three independent experiments (p<0.05; one-way ANOVA). Each independent experiment was performed using ODMs generated from a successive passage of the duodenal 3D organoids and FhNEJ obtained from an independent sub-batch of freshly excysted *F. hepatica* metacercariae. GAPDH and Ezrin band intensities remained stable across experimental conditions, supporting their utility as loading controls in this experimental system (**Supplementary Figure 5F**).

### 2.3 Application of the ODM co-culture model to study epithelial responses to FhNEJ surface-expressed proteins

To further explore the versatility of the ODM platform beyond co-culture with live parasites, differentiated ODMs were stimulated with a tegument-enriched protein fraction obtained from FhNEJ (FhNEJ-Teg). Increasing concentrations of FhNEJ-Teg (0–50 µg/ml) were added to differentiated ODMs and barrier function and junctional organisation were then assessed using the same functional and structural readouts established above. As in the previous experimental set-up, untreated ODMs served as negative controls, and ODMs treated with the apoptosis-inducer staurosporin served as positive controls for epithelial barrier breakdown (Holthaus et al., 2022). Results showed that none of the assayed FhNEJ-Teg concentrations induced detectable alterations in epithelial barrier function, as shown by the retention of physiological TEER (**Figure 4A**) and the normal expression and distribution of the tight junction proteins ZO-1, CLDN-1, and claudin 2 (CLDN-2) as revealed by IFA (**Figure 4B**).

**Figure 4:**
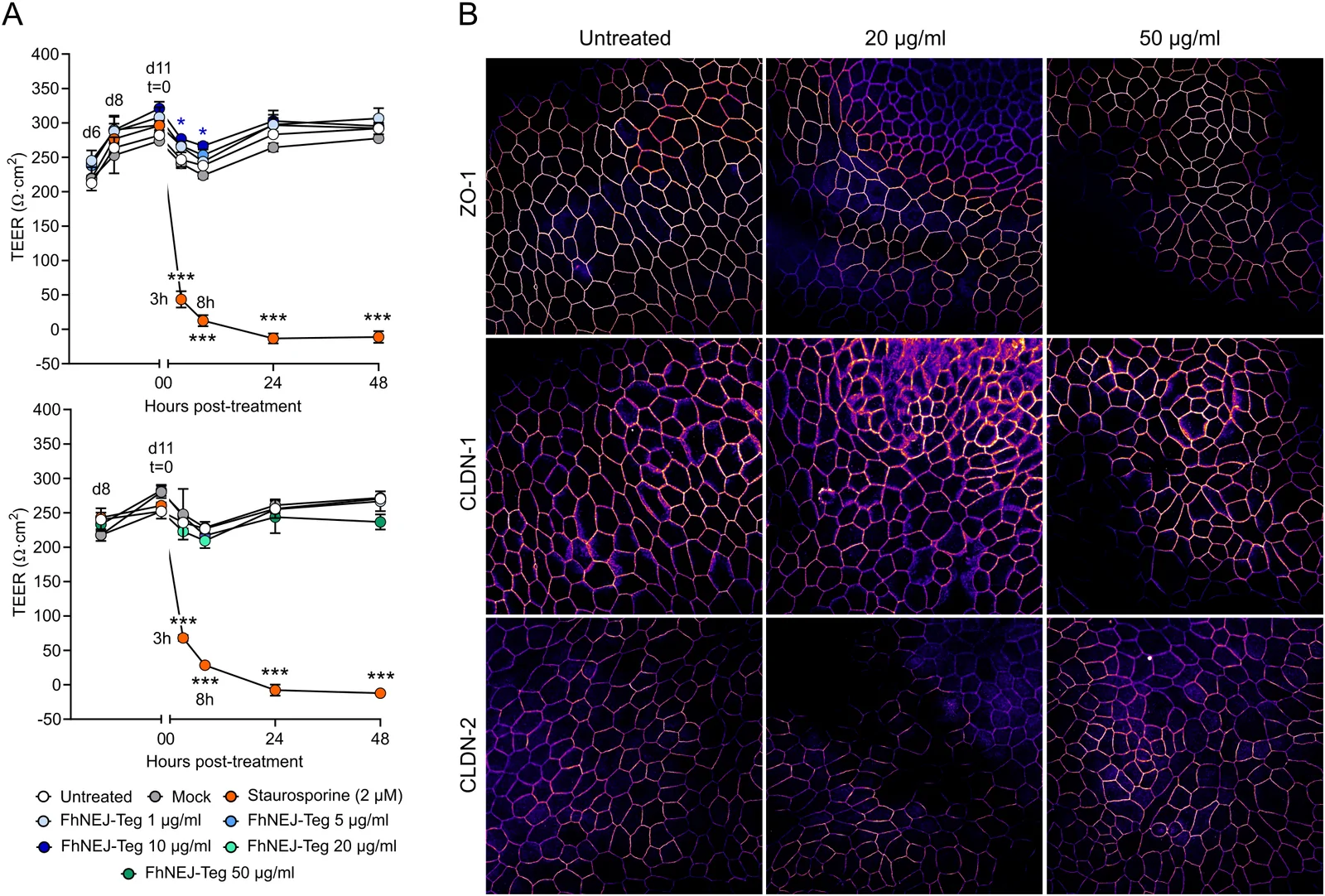
Incubation of ODMs with FhNEJ-Teg does not affect barrier function. **(A)** Time- and dose-dependent evolution of TEER in ODMs treated with increasing amounts of FhNEJ-Teg. Data points indicate the mean of three technical replicates ± SD, and asterisks indicate significant differences between the indicated groups and the untreated condition (*p<0.05; ***p<0.001; one-way ANOVA). **(B)** Representative IFA images of the tight junction proteins ZO-1, CLDN-1, and CLDN-2 at 72 hours after ODM stimulation with 20 µg/ml and 50 µg/ml of FhNEJ-Teg. Images show a single channel displayed with colour-coded intensities. DAPI is shown in greyscale. Specimens were visualised in a Leica Stellaris 8 confocal microscope using the 63× objective. Scale bars, 20 µm.

### 2.4 Compatibility of the ODM–FhNEJ co-culture system with quantitative proteomic profiling

To determine whether the ODM–FhNEJ co-culture system can be used to generate molecular insight into epithelial responses following interaction with FhNEJ, quantitative proteomic profiling was performed on whole-cell lysates obtained at the end of the infection time course (48 h after FhNEJ addition) from control ODMs and co-cultures with either 5 or 50 FhNEJ. A total of 6,827 proteins were identified at a false discovery rate (FDR)≤1%, demonstrating that the platform generates high-quality proteomic datasets suitable for comparative analysis. The raw peak-intensity values obtained through quantitative proteomic analysis are provided in **Supplementary Table 1**.

Comparative analysis further demonstrated that the pipeline was sufficiently sensitive to detect dose-dependent changes in protein abundance, with 26 and 77 differentially expressed proteins (DEPs) identified following incubation with 5 and 50 FhNEJ, respectively (**Figure 5**; **Tables 1**, **2**), mirroring the dose-dependent response that was recorded through functional assays. Notably, 17 of the 26 proteins (65.4%) identified as differentially abundant following stimulation with 5 FhNEJ were also present in the control ODMs vs. 50 FhNEJ dataset, indicating substantial overlap between the molecular responses detected at the two parasite burdens. Most differentially abundant proteins showed increased abundance following parasite exposure, whereas only four proteins were detected at lower abundance in ODMs incubated with 50 FhNEJ and none were reduced following stimulation with 5 FhNEJ. The complete lists of differentially abundant proteins identified under each experimental condition are provided in **Tables 1** and **2**.

**Figure 5:**
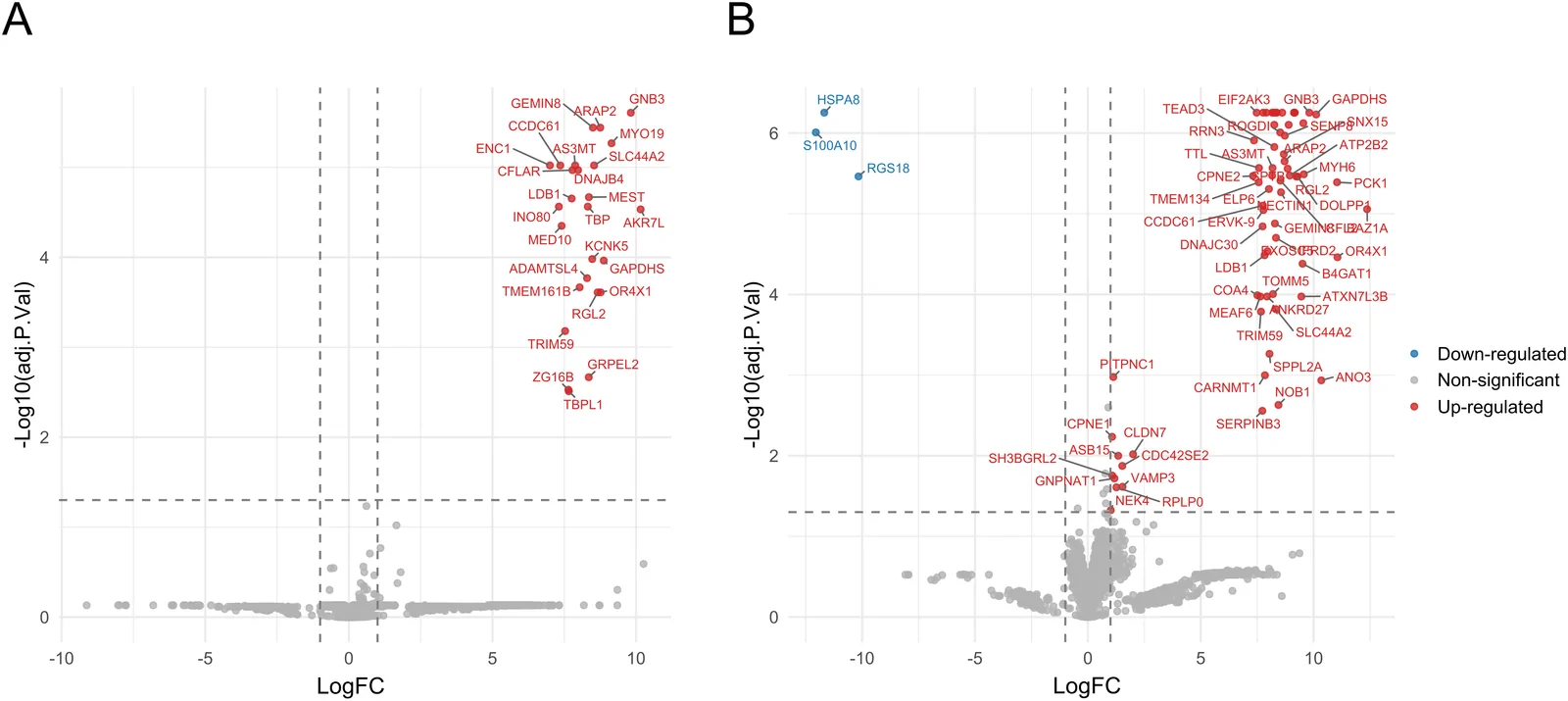
Differentially abundant proteins in ODMs following FhNEJ stimulation. Volcano plots showing the proteins that were detected as differentially abundant in ODMs incubated with 5 FhNEJ **(A)** or 50 FhNEJ **(B)** when compared to untreated controls. The p-value cut-off is set at p ≤ 0.05. The x-axis represents the difference in log1p-transformed intensities between the indicated experimental conditions. For visualization purposes, identities are shown only for proteins with |FC| ≥ 1.

**Table 1:** DEPs in untreated vs. 5 FhNEJ-treated ODMs.

Gene name	Protein name	LogFC	adjPVal
AKR7L	Aflatoxin B1 aldehyde reductase member 4	10.161	2.93E-05
GNB3	Guanine nucleotide-binding protein subunit beta-3	9.819	2.47E-06
MYO19	Unconventional myosin-XIX	9.147	5.39E-06
GAPDHS	Glyceraldehyde-3-phosphate dehydrogenase	8.876	0.000109
OR4X1	Olfactory receptor 4X1	8.768	0.000245
ARAP2	ANK repeat and PH domain-containing protein 2	8.755	3.61E-06
RGL2	Ral guanine nucleotide dissociation stimulator-like 2	8.663	0.000245
SLC44A2	Solute carrier family 44 member 2	8.539	9.51E-06
GEMIN8	Gem-associated protein 8	8.499	3.61E-06
KCNK5	Potassium channel subfamily K member 5	8.475	0.000104
MEST	Mesoderm-specific transcript homolog protein	8.360	2.14E-05
GRPEL2	GrpE protein homolog 2	8.355	0.002151
TBP	TATA-box-binding protein	8.321	2.73E-05
ADAMTSL4	ADAMTS-like protein 4	8.295	0.00017
TMEM161B	Transmembrane protein 161B	8.036	0.000216
DNAJB4	DnaJ homolog subfamily B member 4	7.982	1.07E-05
AS3MT	Arsenite methyltransferase	7.880	9.51E-06
CFLAR	CASP8 and FADD-like apoptosis regulator	7.787	1.07E-05
LDB1	LIM domain-binding protein 1	7.758	2.22E-05
TBPL1	TATA box-binding protein-like 1	7.662	0.003087
ZG16B	Zymogen granule protein 16 homolog B	7.645	0.002964
TRIM59	Tripartite motif-containing protein 59	7.531	0.00066
MED10	Mediator of RNA polymerase II transcription subunit 10	7.410	4.47E-05
CCDC61	Centrosomal protein CCDC61	7.361	9.51E-06
INO80	INO80 complex subunit A	7.314	2.73E-05
ENC1	Ectoderm-neural cortex protein 1	7.001	9.51E-06

A positive fold-change indicates that proteins were over-represented in the ODMs treated with the parasites (red). The opposite is true when the fold-change is negative (none). Proteins shaded in grey were also identified in the untreated vs. 50 FhNEJ-treated comparison (**Table 2**). LogFC indicates the difference in log1p-transformed intensities between the indicated experimental conditions.

**Table 2:** DEPs in untreated vs. 50 FhNEJ-treated ODMs.

Gene name	Protein name	LogFC	adjPVal
BAZ1A	Bromodomain adjacent to zinc finger domain protein 1A	12.367	8.76E-06
OR4X1	Olfactory receptor 4X1	11.056	3.46E-05
PCK1	Phosphoenolpyruvate carboxykinase	11.037	4.06E-06
ANO3	Anoctamin-3	10.341	0.001162
GAPDHS	Glyceraldehyde-3-phosphate dehydrogenase	10.112	5.85E-07
GNB3	Guanine nucleotide-binding protein subunit beta-3	9.807	5.56E-07
MYH6	Myosin-6	9.567	3.23E-06
NRAS	Transforming protein N-Ras	9.543	7.52E-07
B4GAT1	Beta-1,4-glucuronyltransferase 1	9.509	4.16E-05
ATXN7L3B	Ataxin-7-like protein 3B	9.455	0.000106
DOLPP1	Dolichyldiphosphatase 1	9.287	3.45E-06
RGL2	Ral guanine nucleotide dissociation stimulator-like 2	9.230	3.45E-06
GRPEL2	GrpE protein homolog 2	9.164	5.56E-07
TUBB8	Tubulin beta-8 chain	9.150	5.56E-07
STARD5	StAR-related lipid transfer protein 5	9.129	5.56E-07
ATP2B2	Plasma membrane calcium-transporting ATPase 4	8.941	3.35E-06
MYO19	Unconventional myosin-XIX	8.893	7.87E-07
ADAMTSL4	ADAMTS-like protein 4	8.850	2.75E-06
SENP8	Sentrin-specific protease 8	8.723	1.07E-06
ARAP2	ANK repeat and PH domain-containing protein 2	8.712	2.23E-06
SNX15	Sorting nexin-15	8.677	1.82E-06
ICOSLG	ICOS ligand	8.606	5.56E-07
NECTIN1	Nectin-1	8.554	5.4E-06
CFL2	Cofilin-2	8.530	3.88E-06
ROGDI	Protein rogdi homolog	8.518	9.78E-07
NOB1	RNA-binding protein NOB1	8.443	0.002346
VPS72	Vacuolar protein sorting-associated protein 72	8.364	5.56E-07
IFRD2	Interferon-related developmental regulator 2	8.332	1.98E-05
SLC44A2	Solute carrier family 44 member 2	8.327	0.000152
TMEM161B	Transmembrane protein 161B	8.313	5.56E-07
GEMIN8	Gem-associated protein 8	8.284	1.32E-05
TEAD3	TEA domain family member 3	8.257	1.48E-06
TBPL1	TATA box-binding protein-like 1	8.255	7.87E-07
CNPY4	Protein canopy homolog 4	8.231	5.56E-07
TOMM5	Mitochondrial import receptor subunit TOM5 homolog	8.199	9.89E-05
AS3MT	Arsenite methyltransferase	8.179	2.7E-06
OSCP1	Organic solute transport protein 1	8.163	5.56E-07
SPTB	Spectrin beta chain	8.162	3.35E-06
SPPL2A	Signal peptide peptidase-like 2A	8.044	0.000545
ELP6	Elongator complex protein 6	8.020	4.93E-06
EXOSC5	Exosome complex component RRP46	7.955	2.91E-05
ANKRD27	Ankyrin repeat domain-containing protein 27	7.933	0.000106
MED10	Mediator of RNA polymerase II transcription subunit 10	7.900	5.56E-07
CARNMT1	Carnosine N-methyltransferase	7.843	0.001005
LDB1	LIM domain-binding protein 1	7.827	3.27E-05
ERVK-9	Endogenous retrovirus group K member 9 Rec protein	7.775	8.97E-06
TGFBR2	TGF-beta receptor type-2	7.764	5.56E-07
CCDC61	Centrosomal protein CCDC61	7.755	7.89E-06
DNAJC30	DnaJ homolog subfamily C member 30	7.739	1.43E-05
SERPINB3	Serpin B3	7.728	0.002776
TRIM59	Tripartite motif-containing protein 59	7.668	0.000163
MEAF6	MYST/Esa1-associated factor 6	7.635	0.000106
TTL	Tubulin-tyrosine ligase	7.578	2.7E-06
TMEM134	Transmembrane protein 134	7.567	4.07E-06
COA4	Cytochrome c oxidase assembly factor 4 homolog	7.507	0.000102
EIF2AK3	Eukaryotic translation initiation factor 2-alpha kinase 3	7.476	5.56E-07
RRN3	RNA polymerase I-specific transcription initiation factor RRN3	7.356	1.23E-06
CPNE2	Copine-2	7.315	3.39E-06
CLDN7	Claudin-7	2.001	0.00958
VAMP3	Vesicle-associated membrane protein 3	1.534	0.024285
CDC42SE2	CDC42 small effector protein 2	1.524	0.013366
ASB15	Ankyrin repeat and SOCS box protein 15	1.344	0.010009
RPLP0	Large ribosomal subunit protein uL10	1.264	0.024651
GNPNAT1	Glucosamine 6-phosphate N-acetyltransferase	1.175	0.019083
PITPNC1	Cytoplasmic phosphatidylinositol transfer protein 1	1.128	0.001055
SH3BGRL2	SH3 domain-binding glutamic acid-rich-like protein 2	1.079	0.017617
CPNE1	Copine-1	1.072	0.005823
NEK4	Serine/threonine-protein kinase Nek4	1.014	0.047332
SFXN4	Sideroflexin-4	0.904	0.002522
ARL3	ADP-ribosylation factor-like protein 3	0.842	0.026014
POLR2E	DNA-directed RNA polymerases I, II, and III subunit RPABC1	0.795	0.038948
MED15	Mediator of RNA polymerase II transcription subunit 15	0.770	0.016588
PDK1	3-phosphoinositide-dependent protein kinase 1	0.684	0.029425
MRPS12	Small ribosomal subunit protein uS12m	-0.452	0.045035
RGS18	Regulator of G-protein signalling 18	-10.162	3.44E-06
HSPA8	Heat shock cognate 71 kDa protein	-11.677	5.56E-07
S100A10	S100 calcium-binding protein A10	-12.051	9.78E-07

A positive fold-change indicates that proteins were over-represented in the ODMs treated with the parasites (red). The opposite is true when the fold-change is negative (blue). Proteins shaded in grey were also identified in the untreated vs. 5 FhNEJ-treated comparison (**Table 1**). LogFC indicates the difference in log1p-transformed intensities between the indicated experimental conditions.

Proteins identified by the analysis included molecules associated with epithelial junctions, such as claudin-7 (CLDN7) and nectin-1 (NECTIN1); immune signalling, including the inducible T-cell co-stimulator ligand (ICOSL), transforming growth factor β receptor 2 (TGFBR2), and tripartite motif-containing protein 59 (TRIM59); cellular stress responses, e.g. heat shock cognate 71 kDa protein (HSPA8/HSC70); and mechanotransduction, as represented by the TEA domain transcription factor 3 (TEAD3) and spectrin β chain (SPTB). Together, these proteins illustrate the diversity of biological processes that can be interrogated using the ODM co-culture system established in the present work (**Supplementary Table 2**).

## 3 Discussion

The establishment of intimate physical contact between helminths and host epithelia is a prerequisite for successful invasion and the initiation of host responses (González-Miguel et al., 2021). Despite the importance of this early host–parasite interface, physiologically relevant *in vitro* systems enabling direct visualisation of parasite behaviour at the epithelial surface have been lacking. Using the ODM co-culture system, we were able to visualise with precision the stable attachment of FhNEJ to the intestinal epithelium through the ventral sucker, consistent with previous *in vivo* observations (Sangster et al., 2022). High-resolution confocal imaging further revealed that the ventral sucker could simultaneously engage an area encompassing approximately five to six host epithelial cells, illustrating the level of detail that can be achieved using this model. Interestingly, a small number of FhNEJ were observed beneath the epithelial monolayer whilst maintaining their ventral sucker in close contact with epithelial cells. Although the mechanisms and biological significance of this positioning remain beyond the scope of the present study, these observations highlight the capacity of the model to capture parasite localisation relative to the intestinal epithelium, providing a basis for the development of improved models (e.g. biocompatible or plastic-free) that can sustain parasite trans-epithelial migration. Given the partial epithelial detachment observed at the periphery of the transwell inserts, the FhNEJ detected beneath the epithelial cell layer most likely gained access to this location through peripheral regions rather than by actively trans-migrating across the intact epithelium. However, we were unable to directly capture parasite movement into these regions in our limited time-series analysis (**Supplementary Video 1**).

Having determined that intimate parasite–epithelial interactions are established within the co-culture system, we next evaluated whether it could be employed to assess epithelial barrier function during FhNEJ exposure. The intestinal barrier constitutes the first line of defence against luminal microorganisms and is maintained through the coordinated organisation of intercellular junctions, including tight junctions, adherens junctions and desmosomes (Peterson and Artis, 2014). The combination of TEER measurements and fluorescein permeability assays along with IFA and immunoblot analysis of intercellular junction proteins demonstrated that the ODM–FhNEJ co-culture system supports complementary functional and structural assessment of epithelial barrier integrity. Using these readouts, exposure to live FhNEJ resulted in a dose- and time-dependent decrease in barrier function in ODMs as indicated by reduced TEER and increased epithelial permeability to fluorescein compared with untreated controls. Although flux assays are typically interpreted as a proxy for paracellular permeability (Fiorentino et al., 2013; Holthaus et al., 2022), the observed peripheral detachment of the ODM epithelium in the presence of FhNEJ may have contributed to both the reduction in TEER and the increased fluorescein flux, precluding attribution of these changes exclusively to alterations in tight-junction integrity or paracellular transport. That said, increased fluorescein flux coincided with a marked reduction in the apical localisation of ZO-1, a core component of epithelial tight junctions that regulates paracellular permeability (Van Itallie et al., 2009), raising the possibility that FhNEJ may indeed affect this parameter.

Immunofluorescence analysis of tight junctions revealed a strong reduction in ZO-1 signal at the apical domain of epithelial cells in ODMs incubated with 50 FhNEJ. Together with the rather modest reduction in ZO-1 protein abundance detected by immunoblot analysis, these findings suggest that incubation of ODMs with FhNEJ is most likely associated with altered ZO-1 localisation to tight junctions than with substantial loss of protein expression. This interpretation remains a hypothesis based on the combined IFA and immunoblot observations and will require targeted experimental validation to establish whether FhNEJ directly affect ZO-1 localisation or trafficking. In line with this, changes in protein conformation or post-translational modifications induced by the parasites that may affect ZO-1 recognition by the monoclonal antibody used cannot be excluded. Contrasting these results, CLDN-1 localisation to tight junctions appeared unaffected, indicating that the altered ZO-1 staining pattern was unlikely to result from widespread epithelial loss or technical artefacts of immunostaining. A similar phenotype, characterised by reduced ZO-1 but preserved CLDN-1 localisation, was reported in the colonic epithelium of mice with experimental colitis (Poritz et al., 2007), raising the possibility that this pattern might represents a more general epithelial response to stress or injury rather than being FhNEJ-specific.

The mechanisms underlying the epithelial detachment observed in the ODM co-culture system remain unresolved. Given the well-established role of ZO-1 in coordinating cytoskeletal tension and interactions between epithelial cells and the underlying extracellular matrix (Haas et al., 2022), it is tempting to speculate that altered ZO-1 organisation may contribute to the observed detachment phenotype. Equally, the potent proteolytic activity of FhNEJ excretory/secretory (E/S) products (Robinson et al., 2011; Di Maggio et al., 2016; Cwiklinski et al., 2018) raises the possibility that parasite-derived proteases participate in epithelial detachment, which could be addressed using the present co-culture system in future mechanistic studies incorporating recombinant FhNEJ proteases or protease inhibitors. That said, the present study did not formally assess cell viability, hindering discrimination between active modulation of epithelial barrier function by FhNEJ and epithelial damage resulting from parasite contact. The preserved localisation of CLDN-1 at intercellular junctions and the absence of obvious deterioration of the cultures, as demonstrated by cell medium acidification over time, argue against extensive epithelial cell death upon FhNEJ exposure. However, these observations cannot substitute dedicated viability assays. Consequently, whether there is an active modulation of barrier function by FhNEJ through modulation of ZO-1 localisation remains to be experimentally confirmed. Future studies using the ODM–FhNEJ co-culture model established here will help shed light into the mechanisms underlying these observations.

To further demonstrate the flexibility of the ODM–FhNEJ co-culture platform, ODMs were stimulated with a protein extract enriched for proteins derived from the FhNEJ tegument (FhNEJ-Teg), the outermost layer of the parasites that is in direct contact with host tissues (González-Miguel et al., 2021). Under the concentrations tested, FhNEJ-Teg did not elicit the functional and structural alterations observed following incubation with live parasites, as neither epithelial barrier integrity nor the localisation of tight junction proteins was measurably affected. These findings indicate that surface-expressed FhNEJ molecules alone are insufficient to reproduce the effects of live parasites, highlighting that additional factors may contribute to the observed epithelial barrier dysfunction upon incubation with FhNEJ. Such factors could encompass E/S products secreted by live FhNEJ, including FhNEJ-derived proteases, and/or direct physical interactions between the parasites and the tissue. The experiments presented in this work do not allow these possibilities to be distinguished, but rather identify them as candidate mechanisms for future mechanistic studies. These findings are consistent with previous studies using the same *in vitro* system to investigate *Giardia duodenalis* pathobiology, in which barrier disruption occurred only in the presence of live parasites but not upon incubation with parasite lysates or E/S products, suggesting that mechanical sensing of the parasites by host cells is required to elicit epithelial responses (Holthaus et al., 2022; Hagen et al., 2025). Together, these findings illustrate how the ODM–FhNEJ co-culture system can be used to dissect the relative contribution of different parasite-associated stimuli to epithelial responses during early infection.

The compatibility of the ODM–FhNEJ co-culture system with quantitative proteomic profiling further extends the analytical capabilities of the model by providing an exploratory molecular readout of the early host–parasite interface. Proteomic analysis identified candidate proteins involved in epithelial barrier organisation, cell adhesion, immune regulation, and stress responses in ODMs incubated with live FhNEJ relative to the untreated controls. Although these observations require formal validation before biological conclusions can be drawn, they illustrate the capacity of the ODM co-culture model to generate testable hypotheses regarding the molecular events occurring during the earliest stages of *F. hepatica* infection. Consistent with the functional analyses, the magnitude of proteomic reprogramming increased with parasite burden, demonstrating that the platform is sufficiently sensitive to detect dose-dependent molecular responses. Due to the reduced number of DEPs identified in FhNEJ-stimulated ODMs when compared to untreated controls, functional enrichment analysis did not yield any significantly enriched gene ontology terms. However, manual inspection of the dataset identified candidate proteins associated with epithelial junction organisation, immune signalling, cellular stress responses and mechanotransduction. For example, proteins involved in epithelial junction organisation, including CLDN7 and NECTIN1, together with reduced ZO-1 localisation to tight junctions observed by IFA, support the hypothesis that FhNEJ affect the organisation and dynamics of epithelial junctions (Fukuhara et al., 2002; Ding et al., 2012). Although the tight junction protein ZO-1 was identified in our proteomic dataset, it was not selected by feature selection or differential expression analysis, consistent with the view that FhNEJ primarily alter the localisation of this protein to tight junctions rather than its overall abundance. Likewise, the identification of proteins involved in immune signalling, including ICOSL, TGFBR2 and TRIM59, suggests that the platform is capable of capturing epithelial-autonomous pathways potentially associated with epithelial activation and the initiation of immune responses to infection (Kondo et al., 2012; Roussel and Vinh, 2021; Donnelly et al., 2022). Similarly, the differential abundance of proteins linked to cellular stress responses and mechanotransduction, such as HSPA8/HSC70, TEAD3 and SPTB (Harvey and Tang, 2025) raises additional hypotheses regarding the molecular processes accompanying FhNEJ interaction with the intestinal epithelium.

Interestingly, one of the few proteins detected at lower abundance in ODMs following incubation with 50 FhNEJ was HSPA8, a member of the HSP70 family of molecular chaperones. Beyond their well-established role in protein folding, HSP70 family members contribute to the assembly and organisation of epithelial intercellular junctions (Lechuga et al., 2024) by different mechanisms including the regulation of ZO-1 expression and recruitment to tight junctions (Kong et al., 2016; Wang et al., 2018; Yuan et al., 2020). Although a direct role for HSPA8 in regulating ZO-1 localisation has not been demonstrated, the concurrent reduction in HSPA8 abundance and altered ZO-1 organisation observed in the present study raises the hypothesis that these events may be functionally linked. Likewise, the marked reduction in the abundance of S100 calcium-binding protein A10 (S100A10), a plasminogen co-receptor, is consistent with our previous studies suggesting that FhNEJ exploit the host fibrinolytic system to facilitate tissue invasion (Serrat et al., 2023, 2025). Future studies can validate these observations and leverage the ODM–FhNEJ co-culture system to investigate the mechanisms underlying FhNEJ-mediated alterations in tight junction dynamics, the potential exploitation of host-derived fibrinolytic pathways, and the initiation of immune responses to infection, amongst others.

Although highly informative, an important limitation of this study is that all experiments were performed using ODMs derived from a single human donor. Consequently, the extent to which the observed epithelial and proteomic responses are influenced by inter-individual variability remains to be determined. Future studies should therefore assess the reproducibility of these findings across ODMs established from additional human donors, as well as from the natural hosts of *F. hepatica*, including cattle and sheep, to address potential inter-species differences in responses to infection. The adaptation of this experimental approach to different host species could also facilitate comparative studies of the early host–parasite interface from a One Health perspective, which is increasingly recognised as essential for developing effective and sustainable control strategies against multihost neglected zoonotic diseases including fasciolosis (Webster et al., 2016; Mas-Coma et al., 2023). An additional constraint of the ODM system employed in this study is that host cells are seeded on polycarbonate filters that FhNEJ are unlikely to traverse; therefore, ODMs cannot yet fully replace *ex vivo* models that are currently used to study parasite trans-intestinal migration (Becerro-Recio et al., 2022a). In this regard, recently developed membrane-free transwell systems where cells are seeded on hydrogels may provide a valuable *in vitro* alternative to investigate parasite migration across the intestinal wall (Hofer et al., 2025). Moreover, more sophisticated organoid-based models that better recapitulate the intestinal architecture (Nikolaev et al., 2020; Hofer et al., 2025) or variations that incorporate microbiota (Puschhof et al., 2021) and/or immune cells (Lawrence et al., 2024; Recaldin et al., 2024) will be key to fully capture the complexity of host–parasite interactions during early-stage fasciolosis.

In summary, this study establishes human duodenum ODMs as a novel, physiologically relevant and experimentally tractable platform to study the interaction between FhNEJ and the intestinal epithelium. The compatibility of the model with image-based analysis, functional assessment of epithelial barrier integrity and exploratory proteomic profiling provides a framework for generating and testing mechanistic hypotheses within the same experimental system. This model opens a unique experimental window into the ‘point of no return’ of fasciolosis, when the parasite first breaches the intestinal barrier, a transient stage that remains poorly understood yet is central for parasite establishment and disease progression (González-Miguel et al., 2021). By enabling systematic investigation of this critical stage of infection, the ODM–FhNEJ co-culture platform provides a valuable resource for future studies aimed at understanding the molecular basis of *F. hepatica* establishment.

## 4 Materials and methods

### 4.1 *In vitro* excystment of *F. hepatica* metacercariae and extraction of the FhNEJ tegument-enriched protein fraction (FhNEJ-Teg)

*F. hepatica* metacercariae (Italian strain, Ridgeway Research Ltd) were excysted as previously described (Serrat et al., 2023). First, CO_2_ was bubbled through 9 ml of distilled water in a 15 ml conical tube for 30–40 s. Subsequently, 1 ml of freshly prepared 0.2 M sodium dithionite solution (prepared in distilled water) was added to obtain a carbonated solution containing 0.02 M sodium dithionite. This solution was immediately added to 1,200–1,500 *F. hepatica* metacercariae, which were incubated for 1 h at 37 °C under ambient atmospheric conditions. Next, metacercariae were washed three times in distilled water by allowing sedimentation and incubated for up to 5 hours at 37 °C and 5% CO_2_ in 5 ml of excystment medium. Excystment medium consisted of Hank’s balanced salt solution (Sigma) supplemented with 10% lamb bile (obtained from a local abattoir) and 30 mM HEPES (Sigma) at pH 7.4. Every hour after addition of excystment medium, FhNEJ were manually recovered under a stereomicroscope using a 20 μl pipette and transferred to a clean plate containing ODM differentiation medium (Section 4.3), where they were allowed to equilibrate for 1 h at 37 °C in a humidified atmosphere with 5% CO_2_ before addition to ODM cultures. The highest number of FhNEJ were recovered during the first 2 h after addition of the excystment medium. Following the final harvest, FhNEJ were transferred to ODM cultures.

The tegument-enriched protein fraction of FhNEJ (FhNEJ-Teg) was obtained by incubating leftover parasites with 1% Nonidet-P40 (Sigma) prepared in distilled water for 30 min at room temperature with mild rotation, followed by centrifugation for 5 min at 300×g, as previously described (Garcia-Campos et al., 2016). Supernatants containing the FhNEJ-Teg fraction were quantified using the Pierce BCA Protein Assay kit (Thermo Fisher) and stored at −80 °C until use.

### 4.2 Three-dimensional organoid culture

Three-dimensional (3D) organoids from the human duodenum were generated as previously described (Holthaus et al., 2022) and were readily available for the present study. 3D organoid cultures (**Supplementary Figure 1A**) were maintained by passaging once a week at a 1:3 split ratio, as previously described (Holthaus et al., 2022; Warschkau et al., 2022). Briefly, organoids were passaged by centrifugation for 5 min at 4 °C and 350×g, followed by chemical dissociation into single cells using TrypLE Express (Thermo Fisher Scientific) containing 10 µM Y-27632 (ROCK inhibitor; Tocris) for 5 min at 37 °C. Single cells were centrifuged for 5 min at 4 °C and 500×g and resuspended in an appropriate volume of organoid growth medium (see below) mixed with Cultrex basement membrane extract (Bio-Techne) at a 1:2 ratio. Next, 50 µl of cell suspension was seeded into each well of a 24-well plate and incubated at 37 °C for 30 min to allow polymerisation of the basement membrane extract. Cells were cultured in 500 µl of organoid growth medium at 37 °C in a humidified, 5% CO_2_ atmosphere and medium was replaced every two days. Organoid growth medium was prepared as previously described (Holthaus et al., 2022), with minor modifications in composition (Hagen et al., 2025). Specifically, organoid medium consisted of 50% Wnt3a/R-Spondin-3/Noggin (WRN) conditioned medium (CM) (ATCC, CRL-3276), 20% RSpondin-1 CM (kindly provided by Calvin Kuo, Stanford University), 10% mNoggin CM (kindly provided by Hans Clevers, Utrecht University), 50 ng/ml human recombinant EGF (Peprotech), 10 mM HEPES (Thermo Fisher Scientific), 2 mM stable glutamine (Capricorn), 100 μg/ml streptomycin, 100 U/ml penicillin (Capricorn), 1x NCS21 (Capricorn), 250 ng/ml biotin (Roth), 1x N2 (Capricorn), 1 mM N-acetylcysteine (Sigma), 10 mM nicotinamide (Sigma), 500 nM A83-01 (Sigma), and 10 μM SB202190 (Cayman Chemicals) in Advanced DMEM/F-12 (Thermo Fisher Scientific). The Rho kinase inhibitor Y-27632 (Tocris) was added at a final concentration of 10 µM to cell culture medium to inhibit anoikis and was removed the day after seeding.

### 4.3 Generation of two-dimensional organoid-derived epithelial monolayers

Organoid-derived epithelial monolayers (ODMs) were generated from 3D duodenum organoids following established protocols (Holthaus et al., 2022; Warschkau et al., 2022) (**Supplementary Figure 1B**). Specifically, 7-day old 3D organoid cultures were dissociated as described for passaging in Section 4.2 and 5×10^5^ live cells were seeded in cell culture transwell inserts (0.6 cm^2^, 0.4 µm pore size; MilliCell; polycarbonate membrane) placed in 12-well plates and pre-coated with a mixture of Cultrex basement membrane extract (Bio-Techne) and DMEM/F-12 (Thermo Fisher Scientific) at a 1:20 ratio. For every independent experiment, a total of 12 ODMs were seeded. Both the apical and basal sides of the transwell system were supplied with 400 µl and 800 µl, respectively, of ODM differentiation medium, consisting of organoid growth medium (described in Section 4.2) lacking WRN-conditioned medium, A83-01, and SB202190. The Rho kinase inhibitor Y-27632 (Tocris) was added at a final concentration of 10 µM to cell culture medium in the apical side to inhibit anoikis and was removed the day after seeding. ODM differentiation medium was exchanged every two days, and ODMs were incubated at 37 °C in a humidified, 5% CO_2_ atmosphere.

### 4.4 Establishment of the co-culture system

Eleven to twelve days after ODM seeding, three ODMs were stimulated apically with either live FhNEJ (5 or 50 parasites recovered from freshly excysted metacercariae) or with increasing concentrations of FhNEJ-Teg (0, 1, 5, 10, 20, or 50 μg/ml) and incubated at 37 °C in a humidified atmosphere containing 5% CO_2_. Parasite burdens were selected to represent two different infection intensities whilst remaining compatible with the limited availability and high cost of *F. hepatica* metacercariae, thereby allowing a minimum of three independent experiments to be performed. Three ODMs mock-treated with an equivalent amount of media served as negative controls, whereas three ODMs treated apically with staurosporine (Tocris) at a final concentration of 2 µM were included as positive controls for epithelial barrier breakdown, as previously described (Holthaus et al., 2022). Care was taken to ensure that the final volume of cell culture medium in the apical compartment was identical across all experimental conditions.

Following 48 h of stimulation, two ODMs were processed for IFA (one fixed with methanol and one with paraformaldehyde, as described in Section 4.7), whereas the third ODM was used to prepare whole-cell lysates for immunoblotting and proteomic analysis. All three ODMs within each experimental condition were used for longitudinal TEER measurements and for end-point assessment of epithelial permeability using fluorescein flux assays. This experimental workflow was repeated in three independent experiments, each performed using a consecutive passage of ODMs and FhNEJ obtained from an independent sub-batch of freshly-excysted *F. hepatica* metacercariae (**Supplementary Figure 1C**). The viability of FhNEJ was confirmed throughout the 48 h incubation period in all experiments by monitoring active parasite motility under a stereomicroscope.

To prepare whole-cell lysates, cell culture supernatants were carefully aspirated and 200 μl of 2× Laemmli buffer [4% (w/v) sodium dodecyl sulphate (SDS), 20% (v/v) glycerol, 0.004% (w/v) bromophenol blue, 0.125 M Tris-HCl (pH 6.8), 50 mM dithiothreitol (DTT)] supplemented with cOmplete protease inhibitor cocktail (Roche) was added directly to the cells. Lysates were incubated on a shaker at room temperature for 20 min, boiled at 95 °C for 10 min, and stored at −20 °C until use.

### 4.5 Measurement of transepithelial electric resistance

TEER was measured at different timepoints before and after stimulation with FhNEJ using a Millicell ERS-2 Voltohmmeter (Merck-Millipore) equipped with an Ag/AgCl electrode (STX01; Merck-Millipore) on a heating block set to 37 °C. Resistance values from blank (cell-free) transwell inserts were subtracted from raw readings and adjusted to a surface area of 1 cm², as previously described (Holthaus et al., 2022; Hagen et al., 2025). TEER values were either expressed as absolute values or relative to the time of stimulation with FhNEJ (t_0_).

### 4.6 Fluorescein flux assay

Forty-eight hours after stimulation, and prior to fixation or lysis, 400 µl of ODM differentiation medium containing fluorescein (332 Da, Sigma) at a final concentration of 100 µM was added apically and samples of 20 µl from the basal compartment were taken in triplicate at 0 min (before fluorescein addition), 10 min, 20 min, 30 min, and 40 min after addition of fluorescein. Next, 80 µl of milliQ water were added to each medium sample and fluorescence intensity (485 nm/525 nm) was measured in an Infinite M200 PRO plate reader (Tecan Life Sciences). Intensities were calculated relative to the basal values recorded prior to fluorescein addition (0 min, t_0_).

### 4.7 Immunofluorescent analysis and confocal microscopy

Cell culture supernatants from the ODMs were carefully aspirated and the cells were immediately fixed with either 4% paraformaldehyde (PFA) for 1 hour at 37 °C or ice-cold 20% methanol for 40 min at −20 °C. PFA-fixed cells were permeabilised for 20 min at room temperature in 1×Tris-buffered saline (TBS) containing 0.25% (v/v) of Triton X-100 and 0.1 M glycine and both PFA- and methanol-fixed samples were blocked for 1 hour at room temperature with blocking buffer [1×TBS, 0.05% (v/v) Tween-20, 0.02% (v/v) Triton X-100, 3% (w/v) bovine serum albumin, 1% (v/v) normal goat serum, and 0.02% NaN_3_]. Primary antibodies (antihuman ZO-1 raised in mouse, BD Biosciences, cat. nr. 610967, 1:250, methanol fixation; anti-human CLDN-1 raised in rabbit, Life Technologies, cat. nr. 71-7800, 1:100, methanol fixation; anti-human CLDN-2, Life Technologies, cat. nr. 516100, 1:100, methanol fixation) were diluted in blocking buffer and added to the transwell inserts overnight at 4 °C. After three washes in wash buffer (1×TBS containing 0.05% Tween-20 and 0.02% Triton X-100), the corresponding fluorophore-conjugated secondary antibodies produced in goat were diluted 1:250 in blocking buffer containing 10 µM 4′,6-diamidino-2-phenylindole (DAPI) and added to methanol-fixed samples for 1 hour at room temperature. PFA-fixed wells were incubated with blocking buffer containing 1 µg/ml Phalloidin-iFluor 647 reagent (Abcam) and 10 µM DAPI for 1 hour at room temperature to label F-actin filaments and nuclei, respectively. The samples were then washed three times in wash buffer, mounted into glass slides in Fluoromount-G mounting medium (Invitrogen) and imaged in a Leica Stellaris 8 confocal laser scanning microscope (Leica Microsystems) equipped with 405 nm diode laser and Leica white light laser. Imaging was performed with HC PL APO 40×/1.10 W motCORR CS2 and HC PL APO 63×/1.40 OIL CS2 objectives using the LAS X software, version 4.4.0.24861 (Leica Microsystems). Five stacks spanning the full epithelial depth were acquired per transwell insert with a step size of 1 µm and frame averaging of 2. Images were processed using the FIJI software, version 2.15.0 (Schindelin et al., 2012), and the contrast was equally adjusted across experimental conditions for visualisation purposes. To improve visual intensity discrimination across experimental conditions, images were displayed using the Fire lookup table, which colour-codes signal intensity in a single fluorescence channel. In this scheme, higher-intensity pixels are represented in white/yellow and lower-intensity pixels in blue, facilitating contrast detection on a black background.

### 4.8 SDS-polyacrylamide gel electrophoresis and immunoblotting

Twelve µl of whole-cell lysates were loaded into 4–20% Bis-Tris pre-cast gels (GenScript) and separated in MOPS buffer (GenScript) at constant 200 V. Proteins were then transferred onto nitrocellulose membranes (GE Healthcare) in Tris-Glycine transfer buffer containing 2% methanol and 0.01% SDS (v/v) using standard procedures. Transfer quality was validated by Ponceau S total protein staining. Membranes were then blocked for 1 hour at room temperature in 5% (w/v) skimmed milk diluted in phosphate-buffer saline (PBS) and membranes were incubated overnight at 4 °C with primary antibodies (see below) diluted in PBS containing 0.05% Tween-20 (Sigma; PBS-T) and 5% (w/v) skimmed milk (BioRad). Membranes were then washed three times with PBS-T and incubated for 1 hour at room temperature with the respective horseradish peroxidase-conjugated secondary antibodies diluted in PBS-T containing 5% (w/v) skimmed milk (BioRad). After three washes in PBS-T, protein bands were detected by enhanced chemiluminescence using the SuperSignal West Dura Extended Duration Substrate (Thermo Fisher) according to the manufacturer’s instructions. The following primary antibodies and dilutions were used: anti-human ZO-1 raised in mouse, BD Biosciences, cat. nr. 610967, 1:200; anti-human CLDN-1 raised in rabbit, Life Technologies cat. nr. 71-7800, 1:500; anti-human Ezrin raised in mouse, Abcam cat. nr. ab4069, 1:500; and anti-human GAPDH raised in mouse, Poreintech cat. nr. 600004-1-Ig, 1:500.

SDS-PAGE and immunoblotting were performed in technical duplicate and band intensities were quantified using the FIJI software, version 2.15.0 (Schindelin et al., 2012). For each immunoblot, ZO-1 and CLDN-1 band intensities were independently normalised to Ezrin and GAPDH, and the two normalised values were averaged. The resulting values from the two technical immunoblot replicates were subsequently averaged to generate a single value for each independent biological experiment. Therefore, statistical analyses were performed using the three independent biological replicates. Ezrin and GAPDH abundance remained stable across experimental conditions when normalised to each other, validating their suitability as loading controls in this experimental system (**Supplementary Figure 5F**).

### 4.9 Live imaging of ODMs stimulated with FhNEJ

To enable real-time visualisation of FhNEJ interactions with the intestinal epithelium whilst confirming parasite viability, live-cell imaging experiments were performed using transparent transwell inserts with a smaller growth area than those used for the endpoint assays. This configuration improved observability under bright-field microscopy and increased the proportion of the epithelial surface captured during tile-scan imaging. Specifically, 7-day old 3D organoid cultures were dissociated as described for passaging in Section 4.2 and 2.5×10^5^ live cells were seeded in cell culture transwell inserts polyethylene terephthalate membrane (0.3 cm^2^; polyethylene terephthalate membrane, 0.4 µm pore size; CellQART) placed in 24-well plates and pre-coated with a mixture of Cultrex basement membrane extract (Bio-Techne) and DMEM/F-12 (Thermo Fisher Scientific) at a 1:20 ratio. Both the apical and basal sides of the transwell system were supplied with 200 µl and 400 µl, respectively, of ODM differentiation medium (Section 4.3). The Rho kinase inhibitor Y-27632 (Tocris) was added at a final concentration of 10 µM to cell culture medium in the apical side to inhibit anoikis and was removed the day after seeding. ODM differentiation medium was exchanged every two days, and ODMs were incubated at 37 °C in a humidified, 5% CO_2_ atmosphere.

Seven days after seeding the ODMs, *F. hepatica* metacercariae were excysted as described in Section 4.1 and 25 FhNEJ were added to the apical compartment of the transwell system. Live-cell imaging was performed on a Zeiss Axio Observer.Z1 inverted microscope (Carl Zeiss) equipped with an ApoTome structured illumination system. Environmental conditions were maintained at 37 °C and 5% CO_2_ using an Incubator XL multi S2 (Pecon) TempModule S with Heating Unit XL S and CO_2_ Module S (Carl Zeiss). Images were acquired using a Zeiss EC Plan-Neofluar 10×/0.30 objective, a Colibri 7 LED light source, and a HE DsRed filter cube (Carl Zeiss), with an AxioCam 820 mono camera (Carl Zeiss). Time-lapse imaging with tiles 4×4 was performed, acquiring one image every 5 min for approximately 16 hours. Image acquisition and processing were carried out with ZEN software, version 3.8.1 (Carl Zeiss).

### 4.10 Proteomic analysis of ODM whole-cell lysates

Sample preparation. Three samples per experimental condition (untreated, 5 FhNEJ and 50 FhNEJ) were separately analysed by LC-MS/MS. Each sample represented an independent biological replicate, originating from co-cultures obtained from a consecutive passage of ODMs and FhNEJ obtained from an independent sub-batch of freshly-excysted *F. hepatica* metacercariae. Protein concentrations were determined using the Macherey-Nagel Protein Quantification Assay (Macherey-Nagel) and equal amounts of protein were reduced with 2 mM dithiothreitol at 60 °C for 20 min, and alkylated with 5 mM iodoacetamide diluted in 50 mM ammonium bicarbonate at room temperature for 30 min. Next, proteins were purified using the SP3 protocol (Moggridge et al., 2018; Hughes et al., 2019) to eliminate detergents prior to in-solution protein digestion. Purified proteins were digested overnight at 37 °C with 100 ng of sequencing-grade trypsin (Promega) diluted in 50 mM ammonium bicarbonate and the reaction was stopped by addition of 0.1% trifluoroacetic acid.

LC-MS/MS analysis. Liquid chromatography (LC) was performed by loading 200 ng of digested peptides (diluted in 20 µL of 0.1% formic acid) onto an Evotip Pure tip (EvoSep) following the manufacturer’s instructions, followed by separation using the Evosep One system on an analytical column Performance 15 cm×150 μm, 1.5 μm (Evosep). The eluted peptides were ionised in a captive Spray with 1700 V at 200 °C and tandem mass spectrometry analysis (MS/MS) was performed in a TimsTOF fleX mass spectrometer (Bruker) in data-independent acquisition Parallel Accumulation-Serial Fragmentation (diaPASEF) mode.

Data processing and protein quantification. The PASER system (Bruker) was used to send the raw data for subsequent analysis and quantification with DIA-NN v1.8 software (https://github.com/vdemichev/DiaNN). First, an *in silico*-predicted spectral library was built from the SwissProt_Human database using DIA-NN v1.8 and using QuantUMS (high precision) as the quantification strategy. After raw data normalisation and quantification, proteins were filtered at ≤1% false discovery rate (FDR) to ensure confidence in protein identification. The proteomic analysis was carried out at the Proteomics Unit of the Central Support Service for Experimental Research (SCSIE) of the University of Valencia (Spain), a member of the Instituto de Salud Carlos III (ISCIII) ProteoRed Proteomics Platform.

Statistical analysis. Because our proteomic dataset comprised thousands of quantified proteins but only three biological replicates, conventional statistical approaches based on univariate (protein-by-protein) testing are prone to imprecise variance estimates, making statistical inference less reliable. To improve the robustness of differential protein identification under these conditions, comparison of protein expression levels between FhNEJ-treated and control ODMs combined complementary approaches for feature selection, dimensionality reduction and differential expression analysis. Specifically, penalised regression (elastic-net) and Partial Least Squares Discriminant Analysis (PLS-DA) were used to identify and validate condition-specific protein expression patterns in our high-dimensional dataset with three samples per condition (Torang et al., 2019; Ebrahimi et al., 2022; Janizek et al., 2023; Zhang et al., 2023). Differential expression analysis to get fold-change FC and *p*-values for individual proteins was subsequently performed using the empirical Bayes framework implemented in the *limma* package, which uses information across all the proteins to provide more robust variance estimates than conventional Student’s *t*-tests. This method was shown to improve differential protein expression analysis in small-sample proteomic datasets (Kammers et al., 2015; Ritchie et al., 2015).

Prior to analysis, peak intensities of all the conditions were transformed to the logarithm with base *e* using the log1p function in R. Next, a stringent statistical pipeline employing three distinct methodologies was applied to identify differentially abundant proteins with high robustness, as previously described (Pinheiro et al., 2020; Serrat et al., 2025). First, the glmnet package in R was employed to fit an elastic-net regularised regression model to select those variables (proteins) that better explained each experimental condition (Friedman et al., 2010). To refine the model, the nearZeroVar function from the caret package in R was applied to identify variables with near-zero variance, indicating very low variability across observations. These proteins were discarded from subsequent analysis. The values of the regularisation parameters (alpha and lambda) were optimised with the train function of the caret package through cross-validation re-sampling to obtain the regularised model that best fitted our data. The log-transformed expression values of the selected proteins were standardised through Z-score normalisation to ensure direct comparability of protein expression levels across samples, followed by representation in heatmaps (**Supplementary Figures 4A, B**). Second, feature selection by elastic-net was validated through PLS-DA using the mixOmics package in R (Rohart et al., 2017) (**Supplementary Figure 4C**). This package contains the ‘variable importance in the projection’ (vip) function, which estimates the importance of each variable in the projection. When vip>1.5, the influence of the variable on the response is very high (Pinheiro et al., 2020; Serrat et al., 2025). Third, differential expression analysis between selected pairs of experimental conditions was performed using the limma package in R to get FC differences in protein abundance along with the corresponding *p*-values (Ritchie et al., 2015). Only proteins that were selected as condition-specific by both elastic-net and PLS-DA (vip>1.5) and exhibited a significant FC (p-value ≤ 0.05), as calculated by limma, were considered differentially abundant. Graphical representation of this analysis was performed using volcano plots generated in R software with the *ggplot2* and *ggrepel* packages. Raw peak-intensity values obtained for each sample and used for differential expression analysis are provided in **Supplementary Table 1**. The analysis was performed by the Statistics and Omics Data Analysis Unit of the Central Support Service for Experimental Research (SCSIE) of the University of Valencia, Spain.

### 4.11 Statistical analyses

Plots were created, and statistical analyses were performed using Prism 10 software, version 10.5.0 (GraphPad Software). Data points and graph bars represent the mean of three biological or technical replicates. Error bars represent either the standard error of the mean (SEM) or the standard deviation (SD) to reflect the precision of the sample mean as an estimate of the population mean or the variability amongst individual technical measurements, respectively. Comparisons between more than two experimental groups were performed via one-way or two-way analysis of variance (ANOVA) followed by Tukey *post-hoc* analysis for pairwise comparisons. Unless otherwise stated, the differences were not significant. Statistical analysis of the proteomic data is described in Section 4.10.

## Data Availability

The original contributions presented in the study are included in the article/**Supplementary Material**. Further inquiries can be directed to the corresponding author.
