# Supplementary material for: Development of a novel organoid-based two-dimensional system to model the intestinal host–parasite interface in fasciolosis
Source: Front Cell Infect Microbiol. 2026 Sep 25;16:1886545. doi: 10.3389/fcimb.2026.1886545 (PMC13649504; doi:10.3389/fcimb.2026.1886545)
Supplement: Supplementary file 1 [file DataSheet1.pdf]

Supplementary Material

1 Supplementary Figures and Tables

1.1 Supplementary Figures

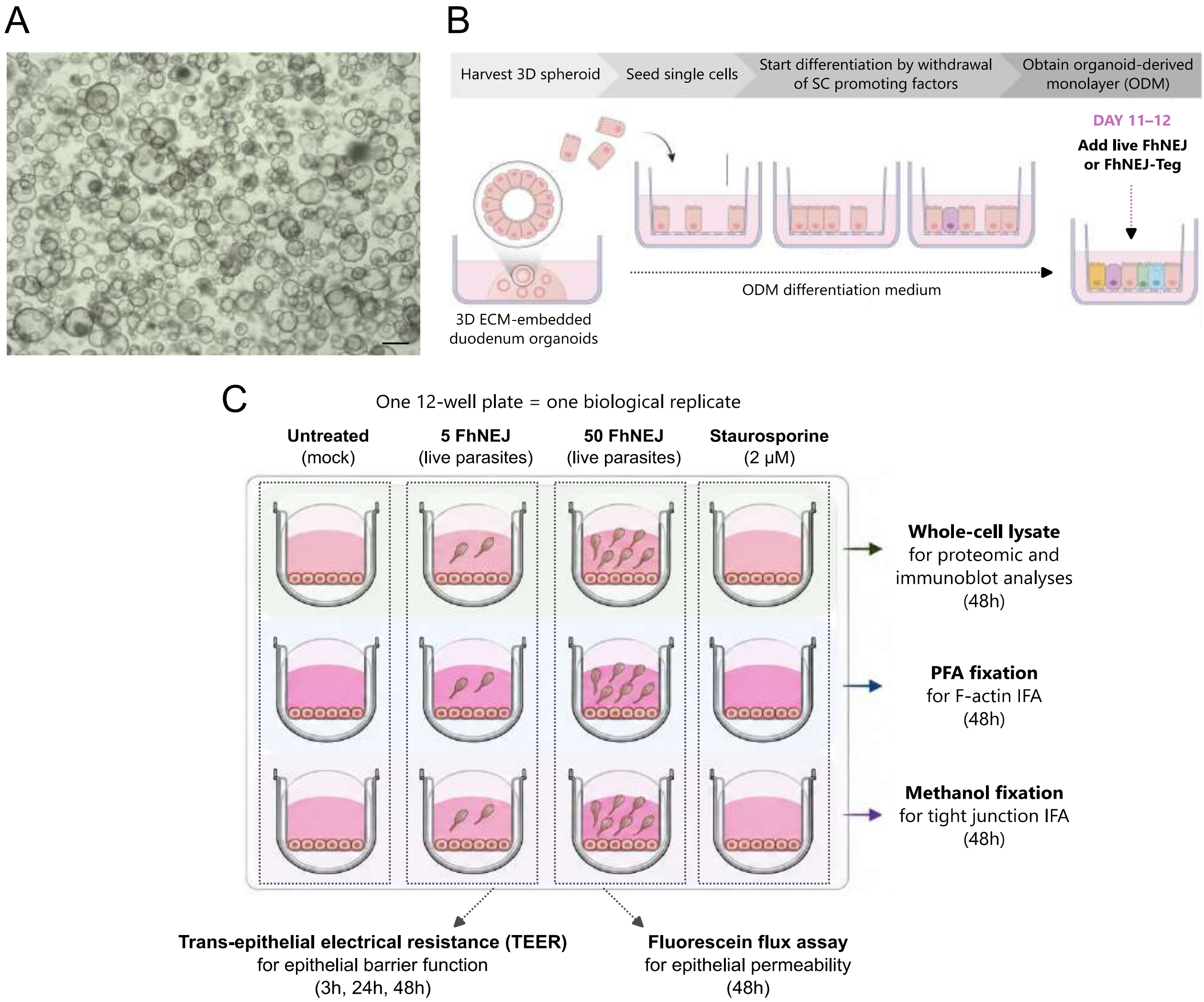

Supplementary Figure 1. Generation of ODMs and experimental set-up. A) Phase contrast image of 3D, stem-cell-enriched organoid culture at passage 23 and 10 days after seeding, derived from human duodenum biopsy samples and used for ODM generation. Scale bar, 200 µm. **B)** Schematic representation of the experimental procedures employed for ODM generation. Modified from Warschkau *et al*., 2022. **C)** Schematic representation of the experimental design used for the ODM–FhNEJ co-culture experiments. For each independent experiment, ODMs were stimulated apically for 48 h with either 5 FhNEJ or 50 FhNEJ. Unstimulated ODMs served as negative controls, and ODMs treated apically with 2 µM staurosporine served as positive controls for epithelial barrier breakdown. Three ODMs were included per condition (technical replicates). All three ODMs were used for TEER measurements at different timepoints and for end-point fluorescein flux assays. Following 48 hours of stimulation, one ODM per condition was processed for whole-cell lysate preparation for immunoblotting and proteomic analysis, while the remaining two were fixed with either paraformaldehyde (PFA) or methanol for immunofluorescence analysis (IFA). The experimental workflow was repeated in three independent experiments (biological replicates). 12-well transwell plate created with ChatGPT.

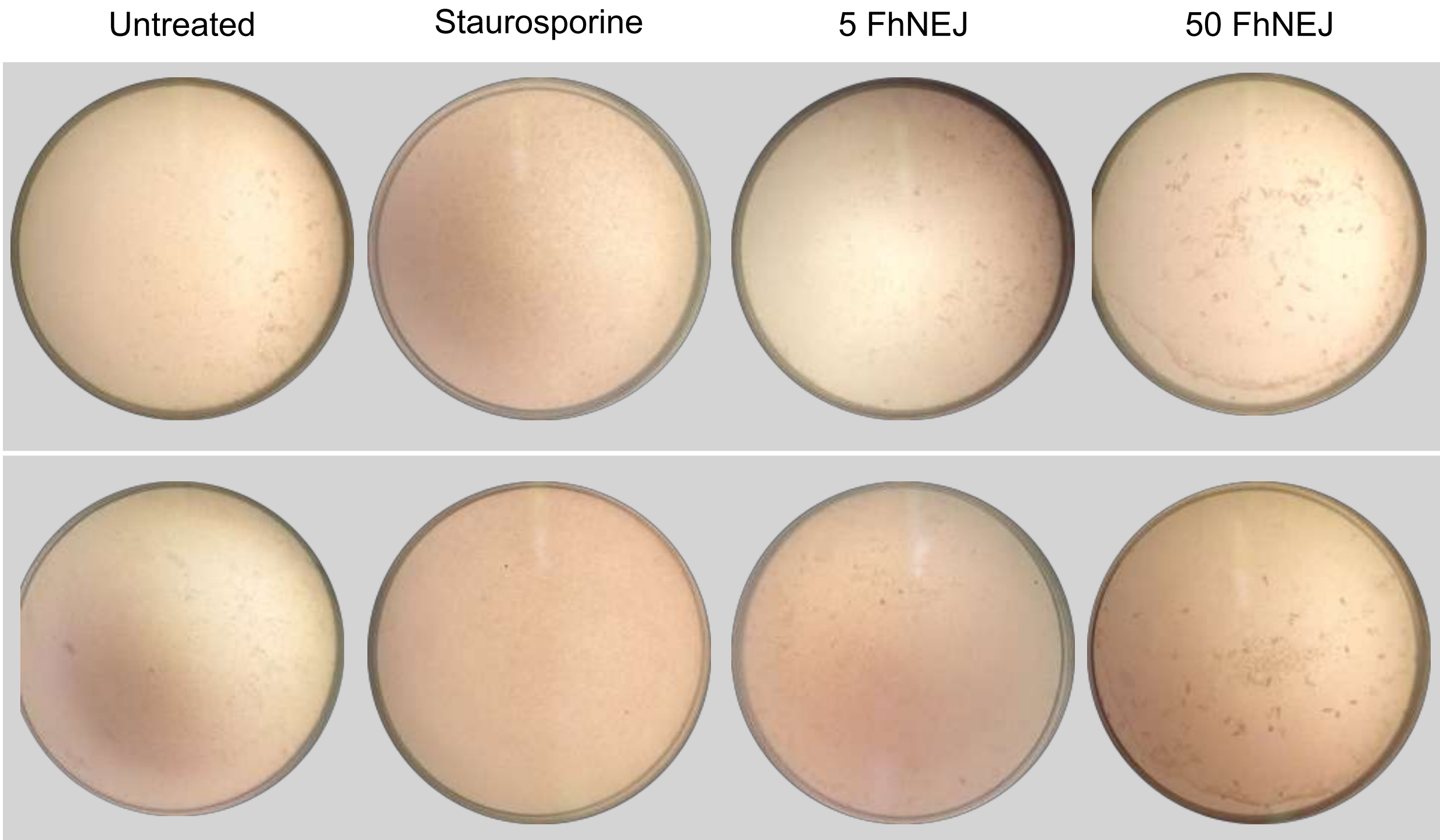

Supplementary Figure 2. Stereoscope images of ODMs after incubation with FhNEJ. Images from ODMs exposed to 5 FhNEJ, 50 FhNEJ, or left untreated at 48 hours after addition of the parasites are shown for two additional independent experiments.

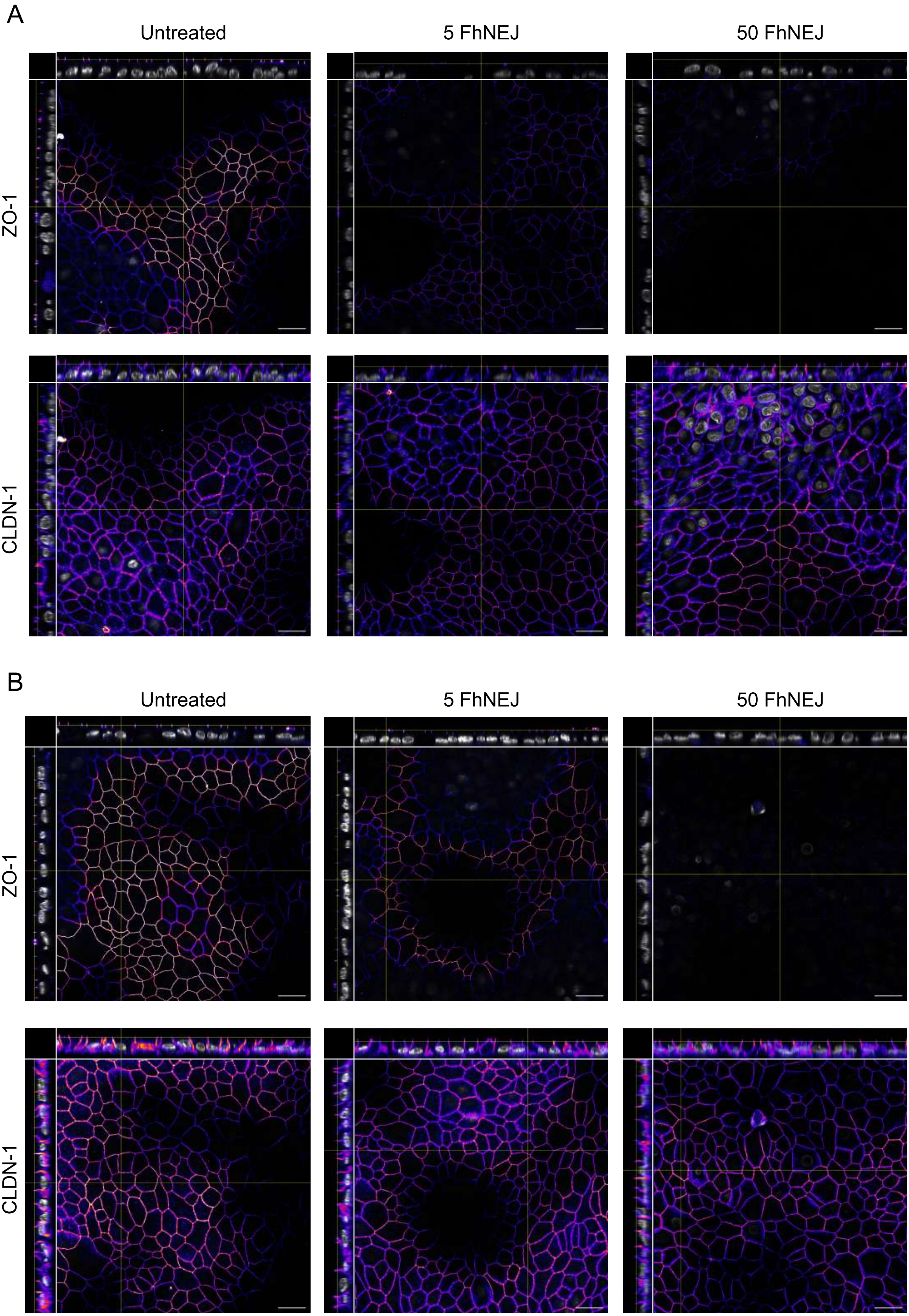
A
Untreated
5 FhNEJ
50 FhNEJ
ZO-1
CLDN-1
B
Untreated
5 FhNEJ
50 FhNEJ
ZO-1
CLDN-1

Supplementary Figure 3 (previous page). Orthogonal stacks of representative IFA images of ZO-1 and CLDN-1 48 hours after addition of FhNEJ. A,B) Specimens from two additional experiments were visualised in a Leica Stellaris 8 confocal microscope using the 63× objective. ZO-1 and CLDN-1 are displayed with colour-coded intensities; DAPI is shown in grey. Scale bars, 20 µm. Each panel displays an independent experiment performed with a consecutive passage of 3D organoids and an independent excystment of a *F. hepatica* metacercariae sub-batch.

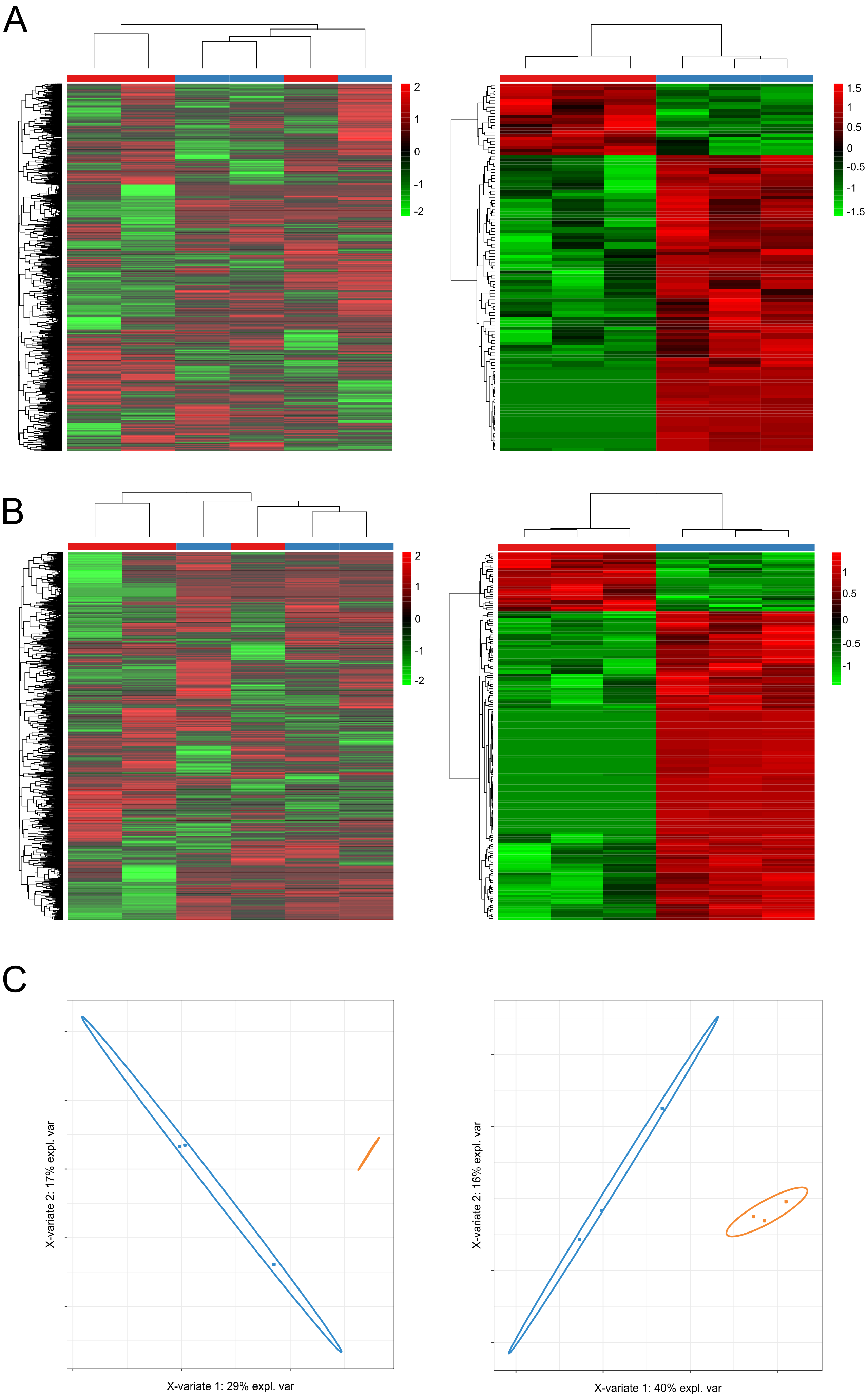
A
2
1
0
-1
-2
1.5
1
0.5
0
-0.5
-1
-1.5
B
2
1
0
-1
-2
1
0.5
0
-0.5
-1
C
X-variate 2: 17% expl. var
X-variate 1: 29% expl. var
X-variate 2: 16% expl. var
X-variate 1: 40% expl. var

Supplementary Figure 4 (previous page). Feature selection by elastic-net and PLS-DA. A,B) An elastic-net penalised regression model was applied to select for proteins that were specifically expressed in each experimental condition. The figures show heatmap representations of Z-score normalised protein expression levels of all the proteins identified (left panels) or proteins that were selected as specific to each experimental condition through elastic-net regularised regression (right panels), for the untreated vs. 5 FhNEJ (A) and untreated vs. 50 FhNEJ (B) comparisons. Red, untreated; blue, ODMs treated with FhNEJ. **C)** PLS-DA was performed to identify the proteins most relevant for distinguishing between experimental conditions. Only those proteins that contributed substantially to the newly projected bidimensional space (as determined by vip>1.5) were considered specific to each experimental condition. Plots are shown for untreated vs. 5 FhNEJ (left panel) and untreated vs. 50 FhNEJ conditions (right panel). Blue, untreated; orange, ODMs treated with FhNEJ.

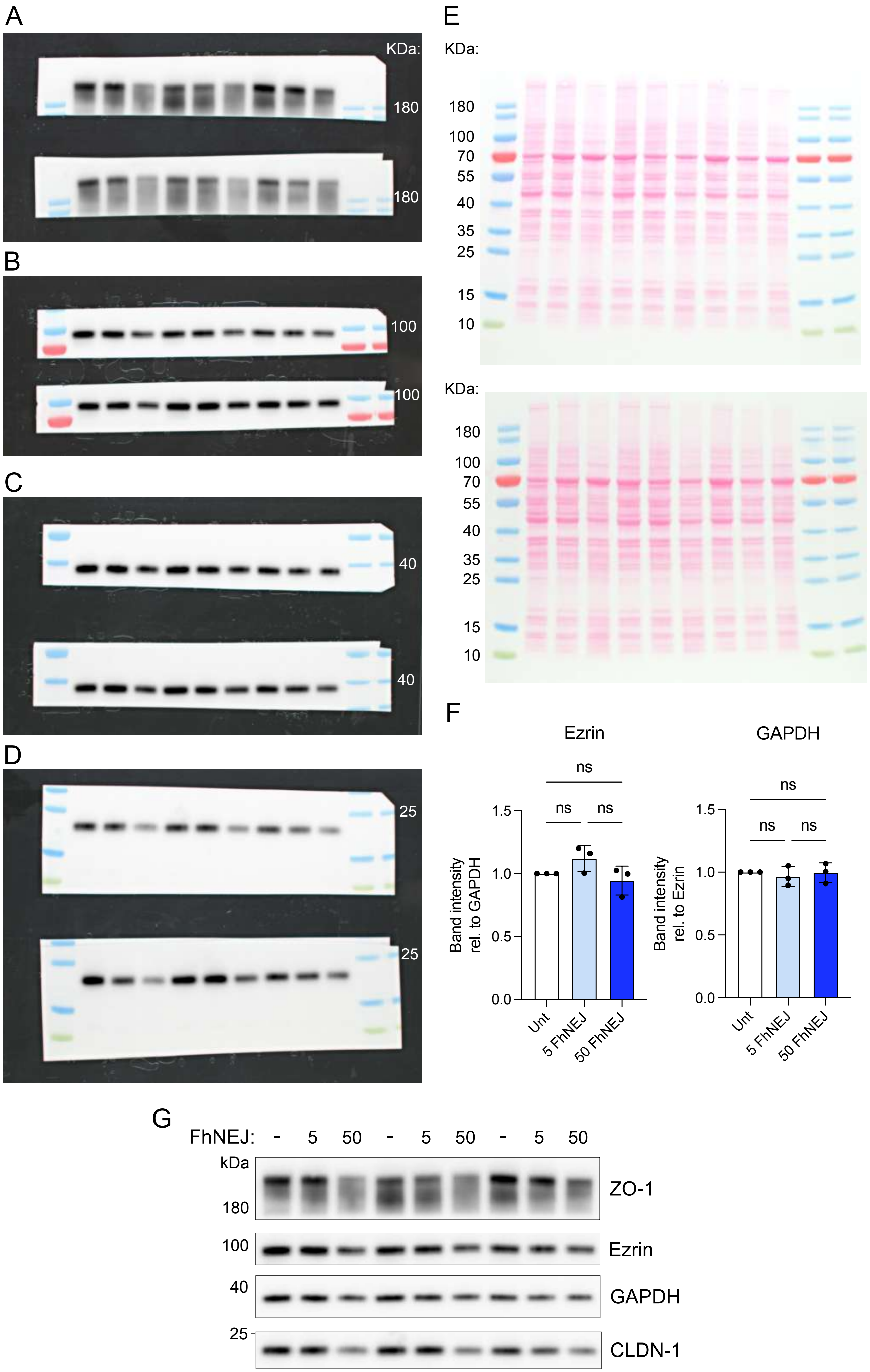
A
KDa:
180
180
B
100
100
C
40
40
D
25
25
E
KDa:
180
100
70
55
40
35
25
15
10
KDa:
180
100
70
55
40
35
25
15
10
F
Ezrin
GAPDH
ns
ns
ns
ns
ns
ns
Band intensity rel. to GAPDH
Band intensity rel. to Ezrin
1.5
1.0
0.5
0.0
Unt
5 FhNEJ
50 FhNEJ
G
FhNEJ: - 5 50 - 5 50 - 5 50
kDa
180
100
40
25
ZO-1
Ezrin
GAPDH
CLDN-1

Supplementary Figure 5 (previous page). Uncropped immunoblot images and assessment of loading control stability across conditions. A–D) Full images of immunoblot membrane sections used for ZO-1 (A), Ezrin (B), GAPDH (C), and CLDN-1 (D) protein detection. Images show the complete membrane sections, including molecular weight markers, and indicate how membranes were sectioned prior to incubation with the corresponding primary antibodies. Two replicate immunoblots are shown, with replicate A displayed above and replicate B (shown in Figure 3B) below. The uncropped images correspond to the same immunoblots presented in the main figure but were acquired using different exposure settings. The exposure displayed in the representative image in Figure 3B was selected to provide optimal visualisation of the immunoreactive bands. Molecular weights are indicated in kDa. **E)** Total protein staining of the uncropped blots with Ponceau S. Top: replicate A; bottom: replicate B (displayed in Figure 3B). **F)** Quantification of Ezrin abundance normalised to GAPDH and GAPDH abundance normalised to Ezrin across experimental conditions. Data points represent independent experiments, each analysed in two technical immunoblot replicates. Bars indicate the mean ± SEM of three independent experiments (ns, not significant; one-way ANOVA). **G)** Second immunoblot replicate used for quantification of the protein bands shown in Figure 3B.

1.2 Supplementary Tables

Supplementary Table 1. Raw peak-intensity values obtained by quantitative proteomic profiling of ODM–FhNEJ co-cultures. Raw peak-intensity values are shown for proteins identified in whole-cell lysates from untreated ODMs and ODMs incubated with 5 or 50 FhNEJ for 48 h. Values are provided for each of the three independent biological experiments analysed. A, untreated ODMs; C, ODMs stimulated with 5 FhNEJ; E, ODMs stimulated with 50 FhNEJ.

Supplementary Table 2. Differentially abundant proteins identified in ODMs stimulated with FhNEJ when compared to their untreated controls, including information about their functions as annotated in the Uniprot database.

2 Supplementary Movies

Supplementary Movie 1. Live imaging of ODMs stimulated with FhNEJ. Time-lapse imaging of ODMs from 0 to 16 hours after addition of FhNEJ showed that the parasites were alive and actively moved throughout the epithelial monolayer. One image every five minutes was acquired in a ZEISS AxioObserver.Z1 imaging system. SpyDNA555 (orange) labelled dead host cells.

Supplementary Movie 2. Video reconstruction of all planes acquired along the entire Z stack of a fixed transwell incubated with FhNEJ, from the apical to the basal side, using a step size of 0.30 µm. The video shows one example of FhNEJ that was recovered under the epithelial monolayer. The specimens were visualised in a Leica Stellaris 8 confocal microscope using the 40× objective. Scale bar, 50 µm.
